# Evolution of rhizobial symbiosis islands through insertion sequence-mediated deletion and duplication

**DOI:** 10.1038/s41396-021-01035-4

**Published:** 2021-07-16

**Authors:** Haruka Arashida, Haruka Odake, Masayuki Sugawara, Ryota Noda, Kaori Kakizaki, Satoshi Ohkubo, Hisayuki Mitsui, Shusei Sato, Kiwamu Minamisawa

**Affiliations:** Graduate School of Life Sciences, Tohoku University, 2-1-1 Katahira, Aoba-Ku, Sendai, 980-8577 Japan

**Keywords:** Soil microbiology, Molecular evolution

## Abstract

Symbiosis between organisms influences their evolution via adaptive changes in genome architectures. Immunity of soybean carrying the *Rj2* allele is triggered by NopP (type III secretion system [T3SS]-dependent effector), encoded by symbiosis island A (SymA) in *B. diazoefficiens* USDA122. This immunity was overcome by many mutants with large SymA deletions that encompassed T3SS (*rhc*) and N_2_ fixation (*nif*) genes and were bounded by insertion sequence (IS) copies in direct orientation, indicating homologous recombination between ISs. Similar deletion events were observed in *B. diazoefficiens* USDA110 and *B. japonicum* J5. When we cultured a USDA122 strain with a marker gene *sacB* inserted into the *rhc* gene cluster, most sucrose-resistant mutants had deletions in *nif*/*rhc* gene clusters, similar to the mutants above. Some deletion mutants were unique to the *sacB* system and showed lower competitive nodulation capability, indicating that IS-mediated deletions occurred during free-living growth and the host plants selected the mutants. Among 63 natural bradyrhizobial isolates, 2 possessed long duplications (261–357 kb) harboring *nif*/*rhc* gene clusters between IS copies in direct orientation via homologous recombination. Therefore, the structures of symbiosis islands are in a state of flux via IS-mediated duplications and deletions during rhizobial saprophytic growth, and host plants select mutualistic variants from the resultant pools of rhizobial populations. Our results demonstrate that homologous recombination between direct IS copies provides a natural mechanism generating deletions and duplications on symbiosis islands.

## Introduction

Symbiotic organisms can co-evolve through adaptive changes in the organization of the functional elements in their genomes. For instance, in animal–bacteria symbiosis, the evolution of obligate symbiont bacteria with intercellular lifestyles has been accompanied by a marked reduction in genome size [1–3]. The best-studied plant–bacteria symbiosis involves legume plants and nitrogen-fixing bacteria called rhizobia [3, 4]. Rhizobia have repeated symbiotic phases (in the plant) and free-living phases (in the soil) [4, 5]. As facultative symbionts, rhizobia generally possess the distinct packages of symbiosis genes (symbiosis islands or symbiotic plasmids) within their genome [3, 4, 6].

Major rhizobia in Alphaproteobacteria include species within the genera *Bradyrhizobium*, *Azorhizobium*, *Sinorhizobium*, *Rhizobium*, and *Mesorhizobium* [7]. Among them, *Bradyrhizobium* species are thought to be ancestral rhizobia, because *Bradyrhizobium*, which shows enormous species diversity (>800 species), nodulates primitive leguminous plants [6, 8, 9].

Rhizobial symbiosis islands on the genomes of *Bradyrhizobium* [10–12], *Azorhizobium* [13], and *Mesorhizobium* [14–18] contain strain-specific symbiotic genes. Comparisons of *Mesorhizobium* genomes reveal different structures of symbiosis islands depending on geography and host plants [16–18]. In *Bradyrhizobium*, symbiosis islands include *nod*, *nif*, and *rhc*, which function in nodulation of roots, N_2_ fixation, and type III secretion system (T3SS), respectively. The symbiosis islands contain conserved regions relevant to the above symbiotic genes and extremely mosaic regions containing insertion sequences (ISs) [10–12, 16–18]. However, little is known about how the symbiosis island structure changes depending on the strain.

ISs are simple mobile genetic elements that impact bacterial evolution including deleterious, neutral, or beneficial effects in bacteria [19]. Multiple copies of an identical IS element dispersed over a genome can promote various genomic rearrangements such as inversion, deletion, duplication, and fusion of two replicons [20, 21]. However, the evolutionary roles of IS elements have been underestimated due to difficulty in their identification on bacterial genomes; (i) repetition of the same ISs on the genomes, and (ii) peculiar signatures of IS elements [22]. ISs are composed of one or two transposase-encoding genes and two terminal inverted repeats, generating two direct repeated sequences as target duplication at the border during transposition [23].

Cultivar-specific restriction of nodulation in soybean has a long history from the 1960s: several dominant genes (*Rj2*, *Rj3*, *Rj4*, *and Rfg1*) in soybeans restrict nodulation with specific rhizobial strains [24–26]. *Rj2*–genotype soybeans restrict nodulation with *Bradyrhizobium diazoefficiens* USDA122 [24, 25]. Rhizobia secrete through type III secretion system nodule outer proteins (Nops), which impact positive, neutral, and negative effects on symbiosis depending on combinations between rhizobia and host plants [27–29]. Although most Nops diminish plant defense responses during rhizobial infection [27–29], *Sinorhizobium fredii* NopP oppositely elicited soybean defense responses [30]. Sugawara and co-workers found that bradyrhizbial NopP is a causal T3SS effector to induce symbiotic incompatibility with *Rj2*–genotype soybeans via effector-triggered immunity [31–33]. Zhao et al. [34] reported adaptive evolution of symbiotic compatibility by IS insertion into type III and *nopP* genes by incompatible combinations between wild-type *Sinorhizobium fredii* and soybeans.

Sugawara et al. [31] also found five mutants that overcame NopP–*Rj2* incompatibility due to partial genome deletions in *Bradyrhizobium diazoefficiens* USDA122. Some of these mutants had deleted T3SS gene (*rhc*) clusters on the symbiosis island; in these mutants the resultant lack of the T3SS machinery prevented NopP secretion [31]. Other mutants, in which both *nif* and *rhc* genes were deleted, showed no N_2_-fixing activity [31] and were parasitic mutants [35].

Here, we studied the dynamics of symbiosis island structures based on IS elements and *Rj2* incompatibility to reveal the mechanisms behind the generation of symbiosis island variations in *Bradyrhizobium*, and their involvement in phenotypic drift for mutualistic and parasitic behaviors.

## Results

### Bioinformatic prediction of IS-mediated deletions

In a previous study, we determined that two ISs in direct orientation on symbiosis island A (SymA) of *B. diazoefficiens* USDA122 were involved in three types of deletions in the USDA122 genome [31]. Here, we examined the IS element distributions in USDA122 SymA to predict and confirm the modes of IS-mediated deletion.

**Fig. 1:**
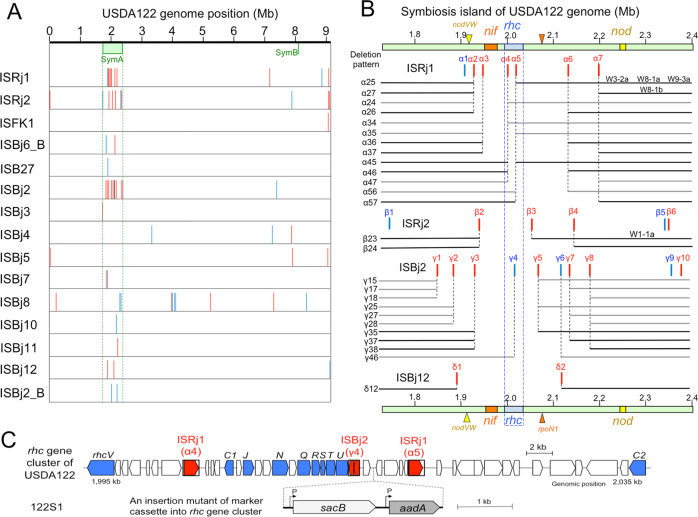
The position of the insertion sequences (ISs) in the genome (A), symbiosis island A (B), and the *rhc* gene cluster (C) of *Bradyrhizobium diazoefficiens* USDA122. Red and blue vertical lines show ISs with their clockwise and anticlockwise directions on the genome, respectively. **A** IS position of 15 different ISs was identified on USDA122 genome. Symbiosis islands of A and B (SymA and SymB) were estimated based on USDA110 genome [10, 11]. Each IS element was numbered along with the location of the SymA, such as α2. **B** Predicted deletion patterns between the ISs were estimated on the assumption that parts of the *rhc* genes encoding the T3SS machinery were deleted between the same IS elements with identical directions on the SymA of USDA122. The deletion patterns were named based on the IS position, such as α25. We included previous deletion patterns of α25, α27, and β23 from the USDA122 mutants W3-2a, W8-1a, W9-3a, W8-1b, and W1-1a [31]. Bold black lines indicate the deletion patterns that were experimentally obtained. Orange, blue, and yellow squares show *nif*, *rhc*, and *nod* gene clusters on SymA, respectively. Yellow and red triangles indicate the positions of *nodVW* and *rpoN1* genes, respectively (see text). **C** Genetic organization of the *rhc* gene cluster on the genomes of *B. diazoefficiens* USDA122 and its marker cassette-insertion mutant 122S1. The marker cassette containing *sacB* (sucrose sensitive gene) and *aadA* (streptomycin and spectinomycin resistant gene) with its own constitutive promoter (P), was inserted in an intergenic region in the *rhc* gene cluster.

The positions of symbiosis islands on the USDA122 genome [36] were determined using the low G + C content of symbiosis islands and their position relative to the Val-tRNA gene in the genome of strain USDA110 [10, 11]. The major symbiosis island SymA (671 kb), which includes *nif*, *rhc*, and *nod* genes, was located in the USDA122 genome at coordinates 1,730,003–2,401,617 bp (Fig. 1A).

We then searched the USDA122 genome for 21 IS elements (ISRj1, ISRj2, ISFK1, IS*1632*, ISBj6_B, ISB27, ISBj2 to ISBj12, ISBj7_B, ISBj5_B, ISBj2_B, and IS*1631*) that were previously identified in the genomes of strains USDA110 [11] and NK6 [37]. When we subjected the USDA122 genome to a BlastN search with the IS elements as query sequences, A total of 63 copies of 15 different IS elements were found, 37 copies of which were located within SymA (Fig. 1A; Table S1).

Seven copies of ISRj1, named α1–α7, and six copies of ISRj2, named β1–β6, resided on SymA on USDA122 genome (Fig. 1B). The transposase genes α2–α7 were oriented clockwise (red in Fig. 1B), whereas α1 was oriented anticlockwise (blue in Fig. 1B). The previously described deletion patterns in USDA122 [31] were designated as α25 (mutants W3-2a, W8-1a, and W9-3a), α27 (W8-1b), and β23 (W1-1a) (Fig. 1B), based on the above nomenclatures for ISRj1 and ISRj2 copies: i.e., α25 is a deletion between α2 and α5, α27 is a deletion between α2 and α7, and β23 is a deletion between β2 and β3.

On the basis of the assumption that the regions harboring *rhc* genes could be deleted between the IS copies in direct orientation by homologous recombination, we found 26 possible deletion patterns mediated by ISRj1, ISRj2, ISBj2, and ISBj12 (Fig. 1B). This suggests that an additional 23 patterns of IS-mediated deletions, other than the previously reported patterns, could occur in USDA122 SymA.

### Deletion mutants of *B. diazoefficiens* USDA122

**Fig. 2:**
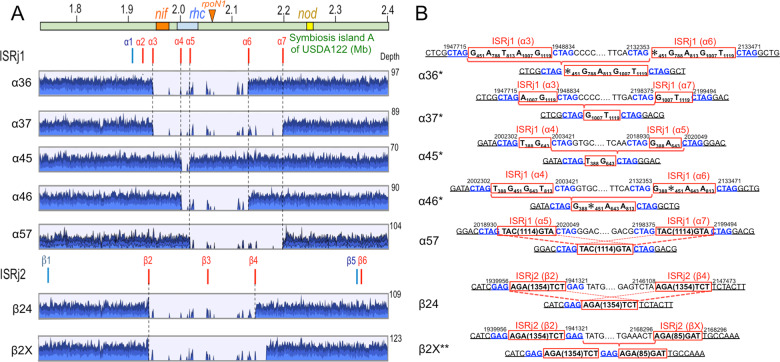
Mapping profiles of MiSeq reads of *B. diazoefficiens* USDA122 mutant on the parent genome (A) and their junction sequences (B). **A** Representative mapping profiles of α36, α37, α45, α46, α57, β24, and β2X were derived from derivatives W17, W29, W27, W46b, HG12, W35, and W20, respectively (Table S2). The colors of IS positions and gene clusters were the same in Fig. 1B. **B** Sequence comparisons of the junctions around the IS elements between the parent (above) and mutant (below) for respective deletion patterns of the USDA122. Red boxes (brackets) and blue letters indicate IS elements and putative target duplication sites, respectively. When DNA sequences of the two IS elements were slightly different, the different nucleotides within the IS elements are shown in the red IS boxes, where “*” shows nucleotide deletion. Red brake lines and solid lines show the estimations of the deletion modes of homologous recombination, by identical and slightly different (asterisked deletion pattern) sequences of the IS elements, respectively. Genomic positions on USDA122 are indicated above the DNA sequences.

To test whether the predicted deletion patterns were generated, we inoculated USDA122 onto 158 plants of *Rj2*-soybean cultivar ‘Hardee’. PCR analyses showed that 30 isolates from 72 spontaneous nodules lost *nifH* and/or *rhcJ* genes but possessed *nodC* gene (Table S2). The remaining 42 isolates showed *nifH/rhcJ/nodC* signals based on PCR analysis, which were not subjected to further genome analyses. However, we continued genome analyses of an isolate HG20 from one nodule of ‘Hardee’ inoculated with 122GFP (Table S3), although HG20 showed positive PCR signals of *nifH/rhcJ/nodC* (Table S2). The mapping of MiSeq reads on the USDA122 genome suggested 6 new patterns of genome deletions (α36, α37, α45, α46, α57, and β24) between ISRj1 or ISRj2 copies in direct orientation (clockwise), and a β2X pattern between an 85-bp short fragment (βX) and full copy (β2) of ISRj2 (Fig. 2A), while the remaining 12 mutants showed the previously identified deletion patterns, α25, α27, and β23 (Table S2).

When the deletion junctions of the isolates were sequenced, the patterns α36, α37, α45, α46, α57, and β24 were confirmed as new deletion patterns (Fig. 2A) that were generated by typical homologous recombination between two copies of the same IS elements in direct orientation (Fig. 2B). Because four ISRj1 copies at positions α3, α4, α6, and α7 on USDA122 SymA possessed minor sequence variations and target duplicates, deletion patterns α36, α37, α45, and α46 consistently kept this sequence variation and a target duplicate (5ʹ-CTAG) during homologous recombination (Fig. 2B). The β24 deletion pattern also showed similar homologous recombination between two ISRj2 copies, at positions β2 and β4, on USDA122 SymA (Fig. 2B). All the deletion patterns, except β2X (Fig. 2A), were predicted in the bioinformatic analysis, validating the prediction method (Fig. 1B).

### Nodulation and N_2_ fixation of USDA122 mutants

**Fig. 3:**
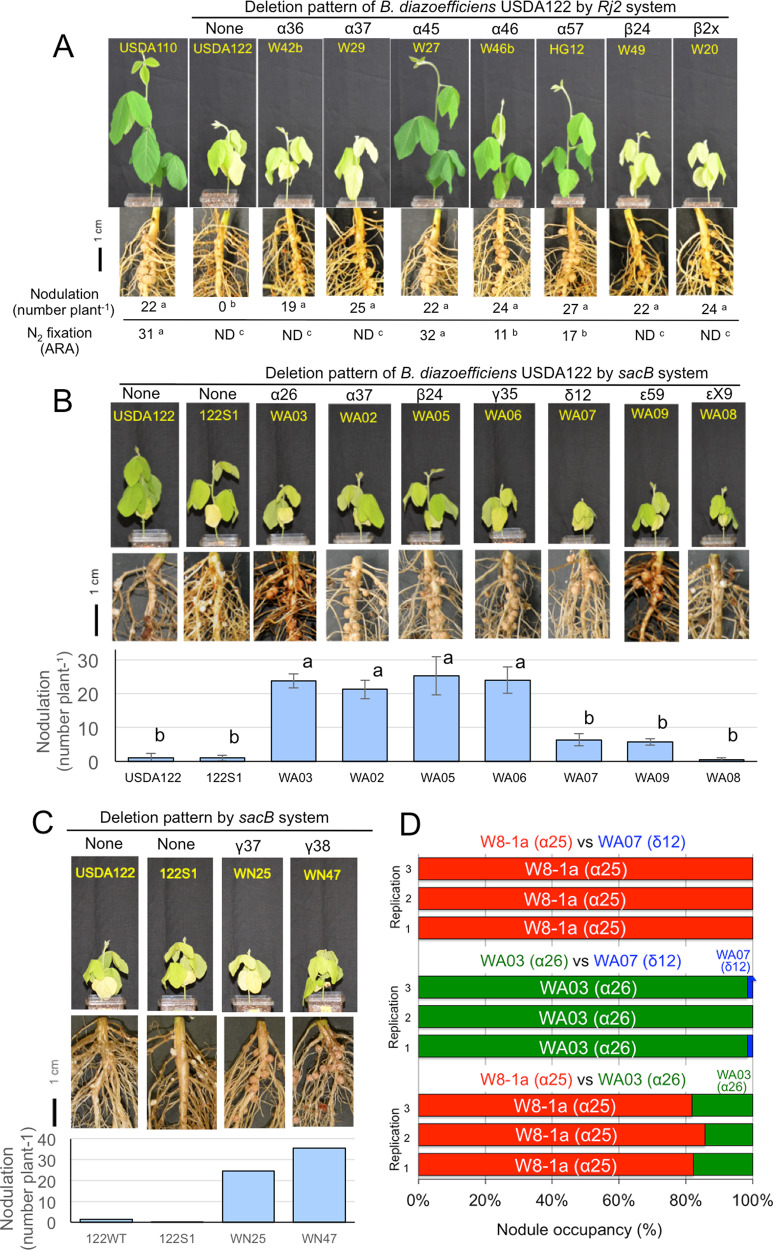
Symbiotic phenotypes of *Rj2*-soybean plants inoculated with representative mutants, for the different deletion patterns of *Bradyrhizobium diazoefficiens* USDA122. *Rj2*-soybean plants were inoculated with deletion mutants by the *Rj2* system (**A**) and by the *sacB* system (**B**, **C**). **A**, **B** “Nodulation” is expressed as the average number of nodules per plant. The bar shows standard deviation. N_2_-fixing activities were evaluated by acetylene-reducing activity (ARA), which is expressed as μmol C_2_H_2_ produced h^−1^ g nodule fresh weight^−1^. ND indicates “not detected” (<0.08 μmol h^−1^ g^−1^). Values are expressed as averages of 3 or 4 replications, except for the nodulation data in panel C (*n* = 2–3). Average values with the same letter are not significantly different by Tukey’s HSD test (*P* < 0.05). **D** Nodule occupancy of *Rj2*-soybean plants inoculated with mixtures of equal amounts of the two deletion mutants. The seeds of *Rj2*-soybean cv. Hardee was inoculated with one-to-one mixed cells of three pairwise combinations: (i) W8-1a (pattern α25) and WA07 (δ12), (ii) WA03 (α26) and WA07 (δ12), and (iii) W8-1a (α25) & WA03 (α26). Nodule occupancy was determined by multiplex PCR (Fig. S8).

Among the seven newly identified deletion patterns (Fig. 2A), *nif* and *rhc* clusters were deleted in α36, α37, β24, and β2X, whereas only the *rhc* cluster was deleted in α45, α46, and α57 (Fig. 2A). To determine the symbiotic phenotypes of representative mutants (W42a, W29, W27, W46b, HG12, W49, and W20) covering the seven deletion patterns were inoculated onto “Hardee”. All plants inoculated with the mutants were well nodulated (Fig. 3A). In contrast, wild-type USDA122 did not form nodules on *Rj2*-soybean roots due to *Rj2*-incompatibility (Fig. 3A). Then, we evaluated the N_2_-fixing activity of the nodules by measuring acetylene-reducing activity (ARA). No ARA was detected in the nodules infected with the mutants W42b (deletion pattern, α36), W29 (α37), W49 (β24), or W20 (β2X) (Fig. 3A), which lacked *nif* genes as well as *rhc* genes (Fig. 2A). ARA was detected in nodules with W27 (α45), W46b (α46), and HG12 (α57) (Fig. 3A), which had a conserved *nif* cluster but deleted *rhc* cluster (Fig. 1B); however, ARA values of W46b (α46) and HG12 (α57) were significantly lower than those of W27 (α45) (Fig. 3A). This finding indicates that genes for efficient symbiotic N_2_ fixation may be located in the regions between α5 and α6 of USDA122 SymA (Fig. 2A). Our survey of this region suggests that *rpoN1* encoding sigma54 of RNA polymerase is a candidate gene for efficient symbiotic N_2_ fixation (Fig. 2A), because the *rpoN1* mutant of *B. diazoefficiens* USDA110 shows reduced N_2_-fixing activity [38].

### Deletion in *B. diazoefficiens* USDA110 and *B. japonicum* J5

**Fig. 4:**
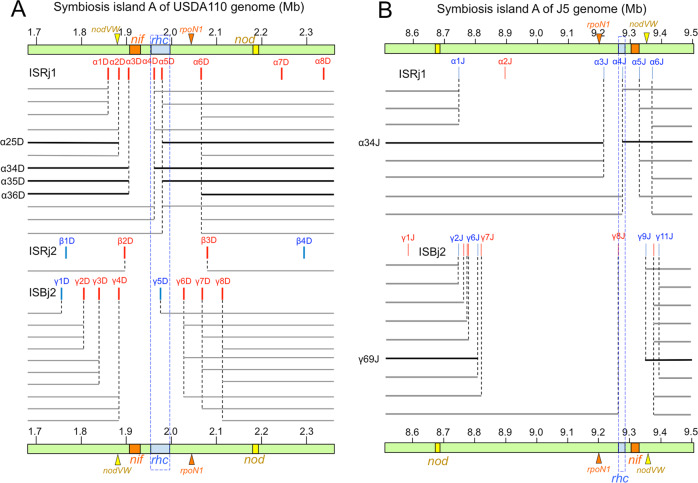
The positions of insertion sequences (ISs) and the predicted IS-mediated deletion patterns within symbiosis island A of *Bradyrhizobium diazoefficiens* USDA110 (A) and *B. japonicum* J5 (B). Red and blue vertical lines show ISs with clockwise and anticlockwise directions on the genomes, respectively. Predicted deletion patterns between the ISs were estimated on the same assumption as in Fig. 1A and B. The deletion patterns were named based on the IS positions, such as α25D for USDA110 and α34J for J5. Other indications are identical to those in Fig. 1.

When IS elements of *B. diazoefficiens* USDA110 [11] and *B. japonicum* J5 [39] were searched using the same strategy as that used for USDA122 above, we identified 16 and 14 different IS elements, respectively, in their genomes (Table S1); these IS elements were concentrated on SymA in both genomes (Fig. S1). Based on the location and orientation of these IS elements and the *rhc* cluster, we predicted 23 and 17 patterns for IS-mediated deletions on SymA of the USDA110 and J5 genomes, respectively (Fig. 4). Although J5 possesses a *nopP* that is incompatible with *Rj2*-soybeans, USDA110 has a *nopP* that is compatible with *Rj2*-soybeans; this gene is slightly different from the USDA122 *nopP* [31]. Therefore, we used a USDA110 derivative that carries a USDA 122–type *nopP* gene (strain 110*nopP*_122_) [31] for the inoculation experiments.

After USDA110 (110*nopP*_122_) and J5 were inoculated onto “Hardee”, we obtained 5 and 3 mutants, respectively, from the spontaneous nodules (Table 1, Table S2). Mapping and sequencing data for these mutants indicated that the deleted regions included *rhc* genes and were between two copies of the same IS (ISRj1 or ISBj2) in direct orientation on SymA (Black bold lines in Fig. 4). The deletion junction sequences in USDA110 and J5 suggested homologous recombination events like USDA122 (Fig. S2).

**Table 1:** Summary of the deletion patterns in mutants of *B. diazoefficiens* USDA122, *B. diazoefficiens* USDA110, and *B. japonicum* J5.

Pattern^a^	Back-ground	IS	Number of mutants		Deleted length (% in SymA)^b^	Symbiotic genes deletion		Plant phenotype^c^	
			*Rj2* system	*sacB* system		*nif/rhc*	Others	Nodulation	N_2_-fixation
α25*	USDA122	ISRj1	12	5	91 kb (14%)	*nif, rhc*		Normal	Fix−
α26	USDA122	ISRj1		11	204 kb (30%)	*nif, rhc*	*rpoN1*	Normal	Fix−
α27*	USDA122	ISRj1	3	3	270 kb (40%)	*nif, rhc*	*rpoN1*	Normal	Fix−
α36	USDA122	ISRj1	9	4	185 kb (27%)	*nif, rhc*	*rpoN1*	Normal	Fix−
α37	USDA122	ISRj1	1	4	251 kb (37%)	*nif, rhc*	*rpoN1*	Normal	Fix−
α45	USDA122	ISRj1	3	1	17 kb (2%)	*rhc*		Normal	Fix+
α46	USDA122	ISRj1	1		130 kb (19%)	*rhc*	*rpoN1*	Normal	Fix+/−
α57	USDA122	ISRj1	1		179 kb (27%)	*rhc*	*rpoN1*	Normal	Fix+/−
β23*	USDA122	ISRj2	2	4	113 kb (17%)	*rhc*		Normal	Fix+
β24	USDA122	ISRj2	3	7	206 kb (31%)	*rhc*	*rpoN1*	Normal	Fix−
γ35	USDA122	ISBj2		5	137 kb (20%)	*nif, rhc*		Normal	Fix−
γ37	USDA122	ISBj2		3	206 kb (31%)	*nif, rhc*	*rpoN1*	Normal	Fix−
γ38	USDA122	ISBj2		1	250 kb (37%)	*nif, rhc*	*rpoN1*	Normal	Fix−
δ12	USDA122	ISBj12		25	227 kb (34%)	*nif, rhc*	*nodVW*	Low	Fix−
ε59	USDA122	Partial ISFK1		1	130 kb (19%)	*nif, rhc*	*nodVW*	Low	Fix−
α34D	USDA110	ISRj1	1		57 kb (8%)	*nif, rhc*		Normal	Fix−
α35D	USDA110	ISRj1	1		74 kb (11%)	*nif, rhc*		Normal	Fix−
α36D	USDA110	ISRj1	1		161 kb (24%)	*nif, rhc*	*rpoN1*	Normal	Fix−
α25D	USDA110	ISRj1	2		96 kb (14%)	*nif, rhc*		Normal	Fix−
α34J	J5	ISRj1	2		61 kb (6%)	*rhc*		Normal	Fix+
γ69J	J5	ISBj2	1		541 kb (54%)	*nif, rhc*	*rpoN1*	Normal	Fix−

^a^Asterisks indicate previous deletion patterns [31].

^b^Percentage of deletion length against symbiosis island A (SymA) of USDA122 (672 kb), USDA110 (681 kb), and J5 (998 kb).

^c^Fix phenotypes were based on Fig. 3 and Fig. S3.

The USDA110 deletions showed four patterns (α25D, α34D, α35D, and α36D) (Fig. 4A, Table S2), all of which deleted both *nif* and *rhc* clusters (Fig. 4A). The J5 deletions showed two patterns (α34J and γ69J) (Fig. 4B, Table S2): in the α34J pattern, only the *rhc* cluster was deleted; but in the γ69J pattern, both *nif* and *rhc* clusters were deleted (Fig. 4B). The pattern γ69J in J5 showed the largest deletion (541 kb), which included 54% of SymA (998 kb) and 5.3% of the entire J5 genome (10.1 Mb) [39].

Symbiotic phenotypes of representative mutants from USDA110 and J5 were also examined by *Rj2*-soybean inoculation (Fig. S3). USDA110 mutants, M7 (deletion pattern, α35D), M8 (α36D), M11b (α25D), and M14 (α34D) showed a negative N_2_-fixing (Fix−) phenotype (Fig. S3A), as expected from their *nif* deletion genotype (Table 1). J5 mutants J2a (γ69J) and J9 (α34J) showed Fix- and positive N_2_-fixing (Fix+) phenotypes, respectively, also in accordance with their *nif* genotypes (Fig. S3B).

### IS-mediated deletion assayed using the *sacB* system

We then examined whether IS-mediated deletions of the USDA122 genome can occur in free-living growth without the *Rj2*-soybean host. Thus, we designed an experimental system (*sacB* system) using a negative selection marker, *sacB*, to detect the deletion of the *rhc* gene cluster (Fig. 1C). A marker cassette containing *sacB* and *aadA* genes with constitutive promoters was inserted into the intergenic region of *rhc* gene cluster of USDA122, resulting in strain 122S1 (Fig. 1C). Because the expression of *sacB* encoding levansucrase is lethal for bacteria in the presence of sucrose [40, 41], we expected that sucrose-resistant colonies would have a deleted or mutagenized *sacB* gene and that a large subset of these colonies would display a deleted *rhc* genes. In contrast, since *aadA* gene confers resistance to spectinomycin and streptomycin (hereafter, Sp/Sm) [42], we expected that colonies resistant to Sp/Sm would have an intact *aadA* gene indicating that the *rhc* cluster was not deleted.

After cultivation of strain 122S1 in HM broth for 5 days, we obtained 32 sucrose-resistant mutants from independent colonies on HM agar plates supplemented with 10% (w/v) sucrose. Of the 32 mutants, 22 were also sensitive to Sp/Sm and produced no *sacB* PCR products (Table 2, Fig. S4), indicating that the marker cassette was likely deleted. Mapping analyses of these 22 mutants on USDA122 genome (Fig. S5A), indicated that 21 mutants were represented in ten patterns of IS-mediated deletions (α26, α36, α37, β23, β24, γ35, γ37, γ38, δ12, and ε59) and one mutant, WA08, was not (εX9) (Table S2). Sequencing verified that the deletion events involved homologous recombination via ISs in the 21 mutants, although the sequence (1081 bp) for homologous recombination of WA09 was not a full sequence of ISFK1 (2592 bp) (Fig. S5B).

**Table 2:** Mutation types of 32 sucrose-resistant mutants in free-living cultures of *B. diazoefficiens* 122S1.

Antibiotics test (Sp/Sm)(Fig. S4)^a^	*sacB* PCR (Fig. S4)	Mutation type	Recombination	Number of mutants	Frequency (10^−4^)^b^	Original data
Sensitive (22 mutants)^c^	Negative (22 mutants)	Marker cassette deletion	Homologous	21 (66 %)	2.2	Fig. S5, Table S4
		Marker cassette deletion	Non-homologous	1 (3 %)	0.1	Table S4
Resistant (10 mutants)	Positive (8 mutants)	*sacB* SNP^*d*^	None	8 (25 %)	0.8	Table S5
	Negative (2 mutants)	Unknown	Unknown	2 (6 %)	0.2	

^a^Spectinomycin (Sp) and Streptomycin (Sm)-sensitive/resistant test on agar plates (Fig. S4).

^b^Frequency calculated from the corresponding mutant number ratio and total frequency of sucrose-resistant colonies of 122S1 that appeared at a frequency of 3.3 ×10-^4^, based on CFU on HM agar plates without sucrose.

^c^Sp/Sm sensitive mutants from the 32 sucrose-resistant mutants: WA01–WA10, WA12–WA19, and WA21–WA24 (Table S4). Among them, 21 were generated from deletions mediated by the full length of ISs, and 1 for a Partial ISFK1 (Table 1).

^d^Single nucleotide polymorphisms (SNP) of the *sacB* gene (Table S5).

Of the 32 sucrose-resistant mutants, ten were resistant to Sp/Sm (Table 2), suggesting the existence of an intact *aadA* gene in *sacB/aadA* cassette (Fig. 1C). Subsequent analyses of *sacB* indicated that 8 of these mutants possessed single nucleotide polymorphisms (SNPs) on *sacB* (Table 2, Table S4), which may lose their levansucrase activity. Combining the two bioassays for sucrose resistance and Sp/Sm sensitivity of 122S1 greatly facilitated the efficient detection of the 21 mutants with IS-mediated deletions. In addition, the frequency of IS-mediated deletion mutants reached 2.2 × 10^−4^, based on total colony-forming units in 5-day-old cultures of 122S1 (Table 2).

To examine the real diversity in the deletion patterns of mutants from free-living cultures, we further isolated an additional 96 sucrose-resistant mutants from 4-day-old cultures of 122S1. Sp/Sm sensitivity assay suggested that 59 mutants had deleted the marker cassette. The IS-PCR analysis indicated that 49 of these 59 mutants (WN01, WN03–WN24, WN26–WN46, WN48, WN49, and WN51–WN53) had already-known deletion patterns (α25, α26, α27, α36, α37, α45, β23, β24, γ35, and δ12) (Table S2). MiSeq reads of the remaining 10 mutants were mapped on the USDA122 genome, and 4 mutants (WN02, WN25, WN47, and WN50) were identified as having new ISBj2-mediated deletion patterns (γ37 and γ38) on USDA122 SymA (Fig. S5. Table 1, Table S2).

**Fig. 5:**
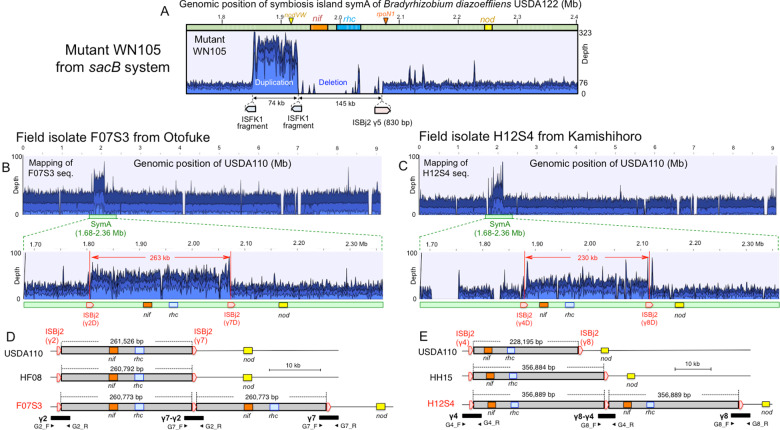
IS-mediated duplication on symbiosis islands of *Brdadyrhizobium diazoefficiens*. **A** Mapping profile of the mutant WN105 from 122S1 on USDA122 symbiosis island, that suggested *nif/rhc* deletions and duplications. (BCDE) Complete genome determination of *B. diazoefficiens* field isolates, carrying IS-mediated tandem duplications, including *nif* and *rhc* genes on the symbiosis island. **B**, **C** Mapping profiles of MiSeq reads of *B. diazoefficiens* isolates of F07S3, from Otofuke field (**B**) and H12S4 from Kamishihoro field (**C**), on the *B. diazoefficines* USDA110 genome. **D**, **E** Structures of tandem duplication in F07S3 (**D**) and H12S4 (**E**) mediated by different copies of ISBj2 on their symbiosis islands, which were verified by PCR sequence analyses (Figs. S10 and S11).

The remaining six mutants (WN101–WN106) showed complicated mapping profiles based on the USDA122 genome (Table S2, Fig. S6). The deletion events likely occurred between a full IS copy (ISRj2 or ISBj2) and a shorter fragment of the corresponding IS in three mutants, WN101, WN102, and WN103 (Fig. S6). MiSeq reads of mutant WN105 showed two characteristic loci of heavily (74 kb) and null (145 kb) mapped regions that were adjacent to each other (Fig. 5A). The heavily mapped region (mapping depth, ~4 times the basal level) had an ISFK1 fragment at each border. The results for mutant WN105 suggest that multiple duplication events likely occurred between two ISFK1 fragments, as well as a deletion event (detected as the 145 kb null mapped region), that included *nif/rhc* gene clusters (Fig. 5A).

### Symbiotic phenotype of deletion mutants isolated by the *sacB* system

Representative mutants from *sacB* system were inoculated onto “Hardee” (Fig. 3B, C). The mutants WA03 (deletion pattern, α26), WA02 (α37), WA05 (β24), and WA06 (γ35) produced large numbers of nodules, in contrast to the parent strain, 122S1. However, WA07 (δ12) and WA09 (ε59) produced significantly lower numbers of nodules than the above mutants (Fig. 3B). In a survey for genes relevant to nodulation, *nodVW* gene, which encodes a flavonoid sensor and regulator [43] was found in the deleted regions of WA07 (δ12) and WA09 (ε59) (Fig. S5A). Thus, it is possible that these mutants were not obtained in the experiments involving inoculation of *Rj2*-soybean ‘Hardee’ plants (hereafter, *Rj2*-system), due to the lack of *nodVW*.

### Comparisons of deletion patterns obtained by the *Rj2* and *sacB* systems

When deletion patterns of USDA122 mutants obtained using *Rj2* system were compared with those from *sacB* system, both systems were found to share common deletion patterns of α25, α27, α36, α37, α45, β23, and β24 (Table 1). However, *sacB* system generated unique deletion patterns (α26, γ35, γ37, γ38, δ12, and ε59) that were not obtained from *Rj2* system (Table 1); 11 and 25 mutants showed the deletion patterns of α26 and δ12, respectively, accounting for approximately half of the 74 IS-meditated mutants from *sacB* system (Table 1). Therefore, we designed an inoculation experiment to compare the competitive nodulation ability of *sacB* system-specific deletion mutants WA03 (α26) and WA07 (δ12) with that of a mutant detected by both systems, W8-1a (α25) (Fig. S7, Table 1).

When one-to-one mixed cells of mutants W8-1a (α25) and WA07 (δ12) were inoculated onto “Hardee”, the nodule occupancy of W8-1a (α25) was extremely dominant against WA07 (δ12) (Fig. 3D). When one-to-one mixed cells of mutants WA03 (α26) and WA07 (δ12) were inoculated, the nodule occupancy of WA03 (α26) was also dominant against WA07 (δ12) (Fig. 3D). These results are expected, due to the low nodulation capability of mutant WA07 (δ12) even in single inoculation experiments (Fig. 3B). When one-to-one mixed cells of mutants W8-1a (α25) and WA03 (α26) were inoculated, the nodule occupancy of W8-1a (α25) was 80%, indicating that nodulation by mutant WA03 (α26) was inferior to that by W8-1a (α25) (Fig. 3D). These results suggest that the mutants with *sacB*-specific deletion patterns α26 and δ12 failed to efficiently nodulate soybean roots under competitive conditions during the selection process of *Rj2* system, thus explaining the lack of mutants with deletion patterns α26 and δ12 obtained from *Rj2* system (Table 1).

### PCR verification of IS-mediated deletion

To obtain more direct evidence for IS-mediated deletions during the cultivation of free-living cells of USDA122 and 122S1, we developed two sets of PCR reactions to detect α25 and α26 deletion events mediated by ISRj1 (Fig. S8). When total DNAs from full growth cultures of USDA122 and 122S1 were used as template DNAs, we detected PCR products of 1.8-kb for the α25 deletion and 2.2-kb for the α26 deletion, in both strains (Fig. S8). This result indicates that free-living cells of USDA122 and 122S1 stochastically generated ISRj1-mediated deletions during simple cultivation.

### IS-mediated duplications on symbiosis islands

Our *sacB* and *Rj2* systems are not designed to detect duplication events on symbiosis islands; however, by chance, a mutant in which both duplication and deletion happened simultaneously, mutant WN105, was identified (Fig. 5A). Therefore, we explored IS-mediated duplications on symbiosis islands by using a culture collection of *B. diazoefficiens* from soybean fields in Hokkaido, Japan. The DNAs of 62 strains from the culture collection were sequenced on the MiSeq platform and mapped to the reference genome of *B. diazoefficiens* USDA110 [11, 44]. Two field isolates, HF07 and HH12, had heavy mapping depths in a region of SymA in the USDA110 genome, when compared with closely related isolates HF08 and HH15 (Fig. S9). When HF07 and HH12 were further purified by single colony isolation on HM agar medium to produce isolates F07S3 and H12S4, respectively, the re-isolates still showed heavy mapping depth in the same region (Fig. 5B, C). Magnification of F07S3 and H12S4 profiles showed that the heavy mapping region spanned 263 and 230-kb of SymA, respectively, and included *nif* and *rhc* genes but not the *nod* genes (Fig. 5B). Interestingly, in both F07S3 and H12S4, the borders of the heavily mapped regions were adjacent to copies of ISBj2: γ2D and γ7D in F070S3 and γ4D and γ8D in H12S4 (Fig. 4A; Fig. 5B). These results suggest that the partial duplication of the symbiosis island on the F07S3 and H12S4 genomes was meditated by ISBj2 copies.

The results of hybrid assembly of MiSeq and Nanopore reads suggest that there was tandem duplication of part of SymA: 260 kb in F07S3 (Fig. 5D) and 357 kb in H12S4 (Fig. 5E). This notion was confirmed by PCR and Sanger sequence analyses targeting the junctions of tandem duplications (Fig. 5D, E; Figs. S10 and S11). The determination of the complete genome sequences showed that the genome sizes of F07S3 (9,432,644 bp) and H12S4 (9,535,585 bp) were larger than those of HF08 (9,109,292 bp) and HH15 (9,177,979 bp), which were obtained from the Otofuke and Kamishihoro fields, respectively, and that this increase in size was due to the tandem duplications, including *nif* and *rhc* genes on SymA (Fig. 5D, E).

## Discussion

The horizontal transfer events of symbiosis islands were mediated by integrative and conjugative elements (ICEs) in *Azorhizobium* and *Mesorhizobium* under laboratory conditions [13, 45, 46]. However, these studies do not address how the variations in symbiosis islands were generated during their evolution between rhizobia and legumes [5]. Symbiosis islands of individual strains of *Bradyrhizobium* have become adapted to leguminous plants [47], suggesting the importance of symbiosis island evolution with host plants [5].

Recombination between IS elements in rhizobia has been known as a mechanism for generating variation in rhizobial genomes. In *Sinorhizobium meliloti* carrying a symbiotic plasmid, cointegrates generated IS-mediated replicon fusion, which did not demonstrate selection for the rearrangements [48]. Zhao et al. [34] reported adaptive evolution of symbiotic compatibility of *Sinorhizobium fredii* by inactivation of *rhc* and *nopP* genes by IS insertion. In contrast, our results demonstrate that homologous recombination between direct IS copies on bradyrhizobial symbiosis island provides a natural mechanism generating deletions, which were verified by the *Rj2* and *sacB* systems on laboratory time scales. Our sequence analysis of the boundary regions of the IS-mediated deletions suggests that the major deletion events occurred via typical homologous recombination [20] between the already existing full IS copies (Fig. 2B, Fig. S2, Fig. S5B). The deletion events also occurred between the fragments of an IS element, ISFK1 (Table 1, Fig. S5B). Thus, IS-mediated deletion does not always require IS transposition by transposase activity. However, we regard the deletion pattern β2X to be the trace of two successive events: ISRj2 transposition and subsequent ISRj2-mediated deletion, based on border sequence analysis (Fig. 2B, Fig. S12).

Most of the IS-mediated mutants with deleted *nif* and *rhc* genes exhibited a Fix- phenotype under the genomic backgrounds of *B. diazoefficiens* USDA122 and USDA110 and *B. japonicum* J5 (Table 1). The incidence of IS-mediated deletion mutants ranged from 2.2 × 10^−4^ (Tables 2) to 8.1 × 10^−4^ in the experiments where 122S1 was grown free-living for 4–5 days. In addition, major deletion events following the patterns α25 and α26 (Table 1) were directly detected in both USDA122 and 122S1 cultures under no selection pressure by PCR (Fig. S8). Thus, these mutants defective in *nif* genes (non-N_2_ fixing mutants) were formed in subpopulations during saprophytic growth (Table 2, Fig. S8) and are likely generated even in soil environments as parasitic bradyrhizobia. However, field isolates of *Bradyrhizobium* from soybean nodules consistently symbiotically fixed N_2_ [49–51]. As for this discrepancy, one explanation may be a host sanction hypothesis to eliminate the parasitic mutants with deleted *nif* genes [5, 52–56]. However, little effect of host sanction was also observed by inoculation tests and modeling in the symbiosis between soybeans and *Bradyrhizobium*, suggesting unknown mechanisms to eliminate non-N_2_ fixing bradyrhizbia in field soils [57].

Previous phylogenetic analyses of a natural population of *Bradyrhizobium* suggested that loss-of-nodulation-capability events were potentially driven by mutations or deletions of symbiosis loci [53, 54]. Here we found that pairs of IS copies in direct orientation were distributed around *nod* gene cluster on SymA of USDA122 (Fig. 1B), USDA110 (Fig. 4A), and J5 (Fig. 4B). Thus, it is possible that IS-mediated deletions of *nod* gene clusters may be an underlying mechanism for the loss of nodulation capabilities in natural bradyrhizobial populations [53, 54].

The symbiosis island structures of isolates F07S3 and H12S4 demonstrated tandem duplications of symbiosis island regions (260–357 kb) via two different copies of ISBj2 in direct orientation (Fig. 5B–E); this process appears similar to IS-mediated deletion in that the duplicated regions contained *nif*/*rhc* gene clusters between two copies of the same IS element in direct orientation. The above two isolates were found among 63 isolates of *B. diazoefficiens* in two independent fields in Hokkaido, Japan. Thus, the duplication events happened in nature, and their incidence was as high as 3% (2/63). Studies of the evolutionary role of IS elements in symbiotic bacteria have focused on genome reduction in obligate symbionts via IS-mediated deletion and host dependence [1–3]. On the basis of our findings, the IS-mediated duplication may play an important role in the evolution of rhizobia as facultative symbionts. Tandem duplications on genomes are of great importance in evolutionary genetics and cancer biology because they can dramatically alter gene functions via subsequent genome remodeling [58–60]. In addition, tandem duplications and deletions are often generated by similar recombination mechanisms [58–60]. Since we found IS-mediated tandem duplications on SymA in two field isolates (Fig. 5B–E), we consider that the structures of bradyrhizobial symbiosis islands are likely in a state of flux, and variants could be generated via tandem duplications and subsequent remodeling, including deletions and genetic exchange within rhizobial populations [58–60]. The structures of large tandem duplications in symbiotic *Bradyrhizobium* species have been previously overlooked due to conventional short DNA sequencing rather than long-read sequencing technologies (ideally > 400 kb).

On the basis of our findings of experimental deletions and natural duplications, we propose a comprehensive hypothesis for symbiosis island evolution: (i) active ISs transpose to form IS-rich loci within low G + C symbiosis islands; (ii) the ISs stochastically induce deletions and duplications that generate variations in the symbiosis islands, and (iii) host plants select the variants of the symbiosis islands in rhizobial populations for improved adaptations.

Extensive attention has been paid to the suppression of plant immunity to establish rhizobia–legume symbiosis [35, 61–64]. However, incompatible NopP protein in rhizobia strongly induces plant immunity via the host *Rj2* allele, leading the host to reject the rhizobial infection [31, 64]. A foliar systemic resistant regulator, glycerol-3-phosphate, is required for *Rj2* incompatibility through root-shoot-root signaling [65]. In addition, Zhang et al. [66] reported that a new resistant protein of *NNL1* gene in soybean accessions directly interacts with NopP effector from *B. diazoefficiens* USDA110 to inhibit nodulation through root hair infection. Taken together with our results, such incompatibility might facilitate co-evolution of symbiosis islands in bradyrhizobia, as a host driving force in nature.

## Materials and methods

Bacterial materials, marker cassette construction, nodulation assay, nitrogen fixation assay, IS identification, PCR primers (Table S5), deletion profile assay, and complete genome determination are described in Supplementary materials.

## Supplementary information

SUPPLEMENTAL MATERIAL
