# Supplementary material for: Evolution of rhizobial symbiosis islands through insertion sequence-mediated deletion and duplication
Source: ISME J. 2021 Jul 16;16(1):112–21. doi: 10.1038/s41396-021-01035-4 (PMC8692435; doi:10.1038/s41396-021-01035-4)
Supplement: Supplementary file 1 — SUPPLEMENTAL MATERIAL [file 41396_2021_1035_MOESM1_ESM.pdf]

Supplementary materials

Materials and Methods

Bacterial strains and growth conditions. Bacterial strains are listed in Table S4. *Bradyrhizobium* strains were grown aerobically at 30 °C in HM salt medium (1) supplemented with 0.1 % arabinose and 0.025 % (w/v) yeast extract. *Escherichia coli* strains were grown at 37 °C in Luria–Bertani medium (2). Agar was added to 1.5 % for solid medium. Antibiotics were added at the following concentrations: for *Bradyrhizobium* strains, kanamycin (Km), 100 mg l^{-1}; spectinomycin (Sp), 100 mg l^{-1}; streptomycin (Sm), 100 mg l^{-1}; and polymyxin B (Px), 50 mg l^{-1}; for *E. coli*, Sp, 25 mg l^{-1}; Sm, 25 mg l^{-1}; and Km, 50 mg l^{-1}. Sucrose was used at 10 % or 5 % (w/v) to counter-select the *sacB*-harboring *Bradyrhizobium* cells.

Construction of a *B. diazoefficiens* derivative harboring the *sacB* marker. The sucrose-sensitive (Suc^s) and Sm/Sp-resistant (Sm^r/Sp^r) marker cassette was generated by PCR to combine a 1,746-bp *sacB* fragment and a 1,123-bp *aadA* fragment that were amplified by PCR from pK18mobsacB and pHP45-omega, respectively. Two chromosomal fragments of *B. diazoefficiens* USDA122 (coordinates 2015413–2016412 and 2016454–2017567) and the Suc^s–Sm^r/Sp^r cassette were combined by PCR to correspond to the chromosomal *rhc* region, whose segment (coordinate 2016413–2016453) was replaced with the cassette, and cloned into pK18mob. The resulting plasmid was transferred into *B. diazoefficiens* USDA122 by triparental mating with the helper plasmid pRK2013. A Px^r Sm^r Sp^r transconjugant was selected, and the correct insertion of the marker cassette on the chromosome was checked by PCR, yielding strain 122S1. PCR primers are listed in Table S5.

Plant inoculation and acetylene reduction assay. Seeds of the soybean cultivar *Glycine max* (L.) Merr. cv. Hardee (*Rj2*) were surface-sterilized and sown in sterile vermiculite with a nitrogen-free plant nutrient solution (3) in a 27-L container or in a 300-ml plant box. *Bradyrhizobium* cell suspension (1×10^9 cells) was added onto the seeds. Plants were grown for 21 to 23 days with a photoperiod of 16-h light at 25 °C and 8-h dark at 20 °C. For acetylene reduction activity, acetylene gas was injected to

10 % (v/v) in a sealed vial containing the root system. The ethylene concentration of the headspace gas was determined using a Shimadzu GC-18A gas chromatograph equipped with a flame ionization detector and a Porapak R column.

Isolation of *Bradyrhizobium* strains from nodules. Nodules were surface-sterilized in 70 % ethanol for 30 s and 1 % (w/v) sodium hypochlorite for 5 min, washed several times with sterile water, transferred into a 1.5 ml tubes containing 50 µl of sterile water, homogenized with a sterile toothpick, and streaked onto HM agar medium. A colony that appeared after 8 d was transferred for purification.

Identification of ISs in the *Bradyrhizobium* genome. *Bradyrhizobium* genome sequences (accession numbers CP013127, BA000040, and CP017637 for USDA122, USDA110, and J5, respectively) were searched by blast using GenomeMatcher (4) with 21 bradyrhizobial ISs deposited in ISFinder database (https://www-is.biotoul.fr) as queries, with a cutoff of ≥ 99 % sequence identity and a ≤ 20 bp length difference.

Identification of a deletion in the *Bradyrhizobium* genome. A cell lysate used as a template for PCR was prepared by treating *Bradyrhizobium* cells with proteinase K (1 mg ml^{-1}) for 20 min at 60 °C, and for 5 min at 95 °C in BL buffer (40 mM TrisHCl, 1 % Tween20, 0.5 % Nonidet P-40, 1 mM EDTA, pH 8.0). PCR was performed using the primers listed in Table S2 and Blend Taq Plus (TOYOBO, Osaka, Japan), using the following program: 2 min at 94 °C, followed by 25 cycles of 30 s at 94 °C, 30 s at 55 °C and 40 s at 72 °C. For the whole-genome resequencing, total DNA was extracted using an Illustra bacteria genomicPrep Mini Spin Kit (GE Healthcare UK Ltd., Buckinghamshire, UK) and was processed using a Nextera DNA Sample Preparation Kit (Illumina, San Diego, CA, USA) to generate a shotgun library with unique index adapters. The library was sequenced on a MiSeq system (Illumina), yielding ~250 bp paired-end reads. The raw reads were trimmed and mapped to reference genomes of *B. diazoefficiens* USDA110^{T} (5), *B. diazoefficiens* USDA122 (6), or *B. japonicum* J5 (7) using CLC Genomic Workbench software (CLC bio, Inc., Aarhus, Denmark) with the following parameters: mismatch cost, 2; insertion cost, 3; deletion cost, 3; length fraction, 0.9; and similarity fraction, 0.9. Deletions detected in the whole-genome resequencing were confirmed by PCR using the primers listed in Table S3 and

sequencing of the PCR products using a 3130 xl Genetic Analyzer (Thermo Fisher Scientific, MA, USA). *B. diazoefficiens* USDA122 and 122S1 cells grown in HM liquid medium were searched, for an ISRj1-flanked deletion by PCR, using primers a2M-F, a5M-R and/or a6M-R1 (Table S5), using the following program: 2 min at 94 °C followed by 40 cycles of 30 s at 94 °C, 30 s at 60 °C, and 2.5 min at 72 °C.

Competitive nodulation. Cell suspensions (1×10^5 cells) were prepared from 5-day-old -cultures of mutants W8-1a, WA03, and WA07. Equal amounts of W8-1a and WA07 cells was mixed and inoculated onto soybean Hardee seeds (2×10^5 cells seed^{-1}). Likewise, the other two sets of cells (W8-1a & WA03 or WA03 & WA07) were also inoculated. After plant cultivation for 4 weeks, the nodules were sampled. DNAs from respective nodules were analyzed by PCR, with the primers listed in Table S5 to discriminate between the three mutants.

Determination of complete genome sequences. Short-read sequencing by Illumina MiSeq platform described above, and long-read sequencing using Oxford Nanopore Technologies (ONT), were used to obtain complete genome sequences of *B. diazoefficiens* strains HH15, H12S4, HF08, and F07S3. Libraries for ONT sequencing were prepared with Native Barcoding Expansion kits (EXP-NBD104) and a Ligation Sequencing kit (SQK-LSK109) and were used on a GridION device with an R9.4.1 flow cell. Filtlong version 0.2.0 (https://github.com/rrwick/Filtlong) was used for quality trimming of the Illumina reads. Sickel-trim version 1.33 (https://github.com/najoshi/sickle) was used for quality trimming of the ONT reads. Hybrid assembly of the Illumina and ONT reads was conducted by Unicycler version 0.4.8 (8). In the cases of H12S4, F07S3, and HF08, assembling was finished manually using some ONT reads that overlapped with terminal sequences of the contigs; the assemblage was verified by PCR analyses and Sanger sequencing, as described in Figs. S1 and S2. The resulting single circular contig was further re-polished with Illumina reads by a variant detection tool of CLC Genomics Workbench and GENETYX-MAC software v. 18.0.3 (Genetyx Co., Tokyo, Japan). The complete genome sequences were automatically annotated using the DDBJ Fast Annotation and Submission Tool pipeline with the standard settings (9). Accession numbers of the complete genomes are AP022638 (F07S3), AP022639 (H12S4), AP022640 (HF08), and AP022641 (HH15).

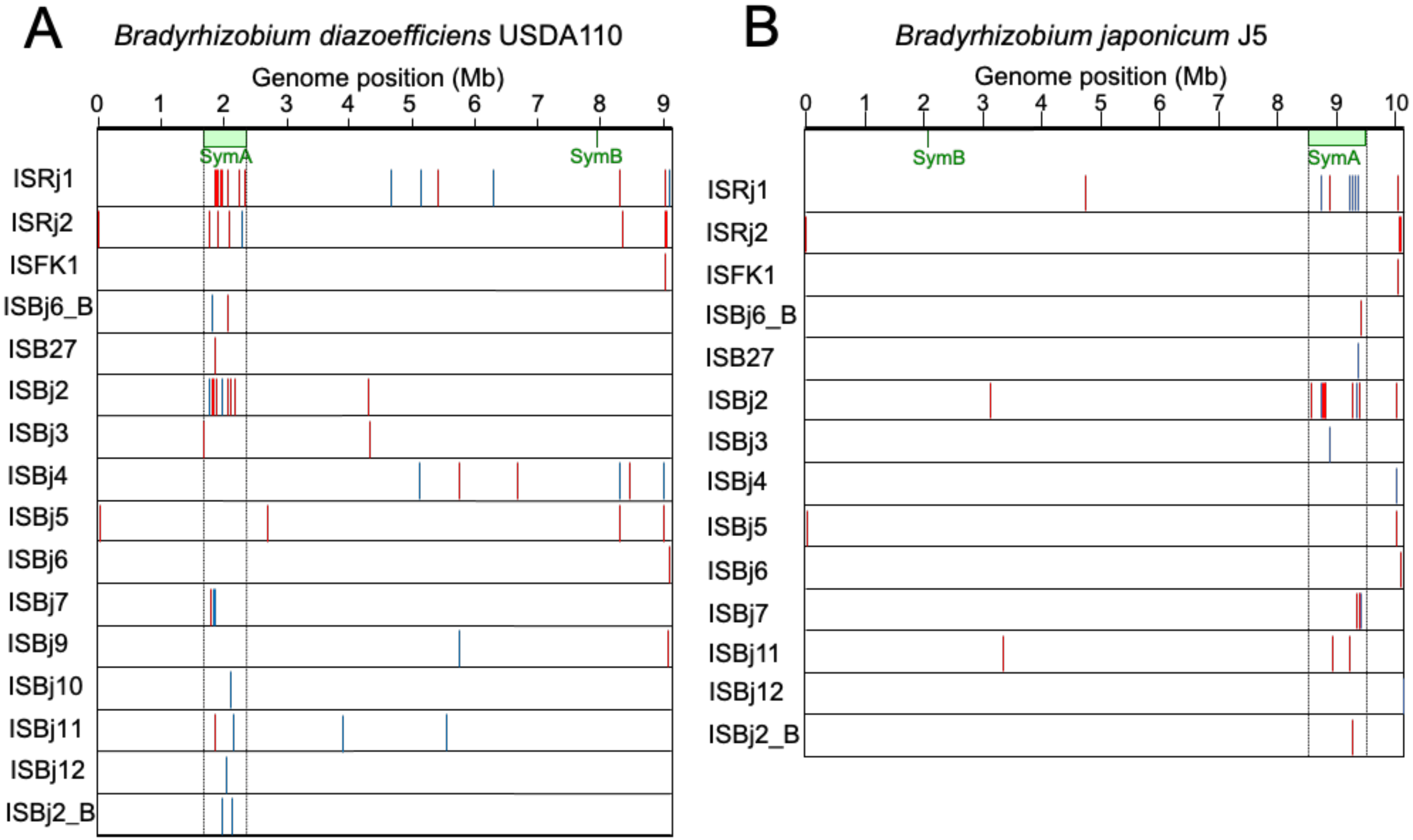

Fig. S1. Position and direction of the insertion sequences (ISs) on the genome of *Bradyrhizobium diazoefficiens* USDA110 (A) and *B. japonicum* J5 (B). Green boxes show symbiosis islands A and B that were estimated from the genome comparisons with the USDA110 genome (see text) (5, 10). Red and blue vertical lines show clockwise and anticlockwise IS directions, respectively. IS nomenclatures were based on those from a previous paper (11).

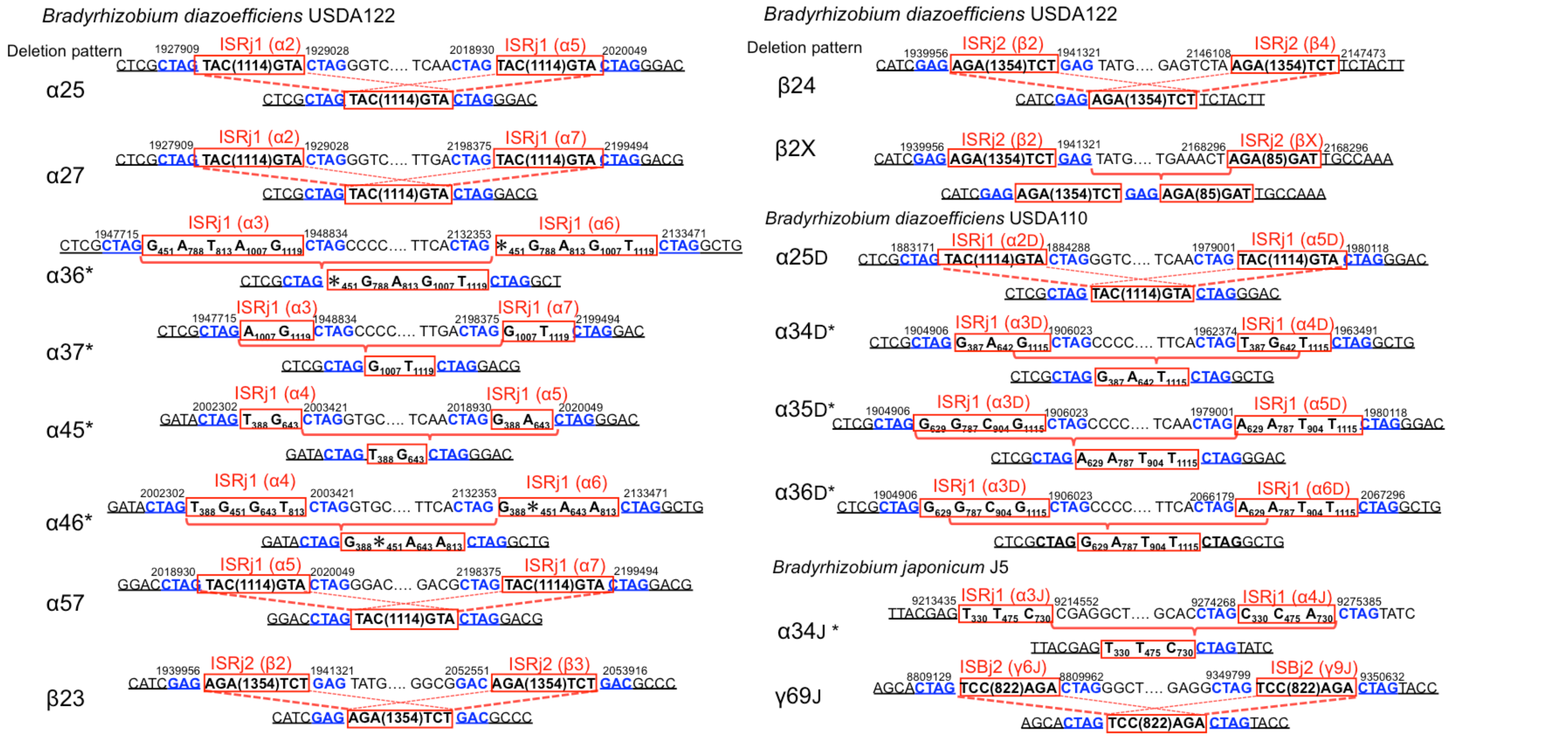

Fig. S2. Sequence comparisons of all junctions around the IS elements between the parent (above) and mutants (below), for respective deletion patterns of *Bradyrhizobium diazoefficiens* USDA122, USDA110, and *B. japonicum* J5. Red boxes and blue letters indicate IS elements and putative target duplication sites, respectively. When DNA sequences of two IS elements are slightly different, the different nucleotide positions within the IS elements are shown in the red IS boxes, where "＊" shows nucleotide deletion. Red solid and brake lines show the estimations of the deletion modes of the homologous recombination, by identical and slightly different (asterisk) sequences of the IS elements, respectively. Genomic positions on USDA122, USDA110, and J5 are indicated above the DNA sequences.

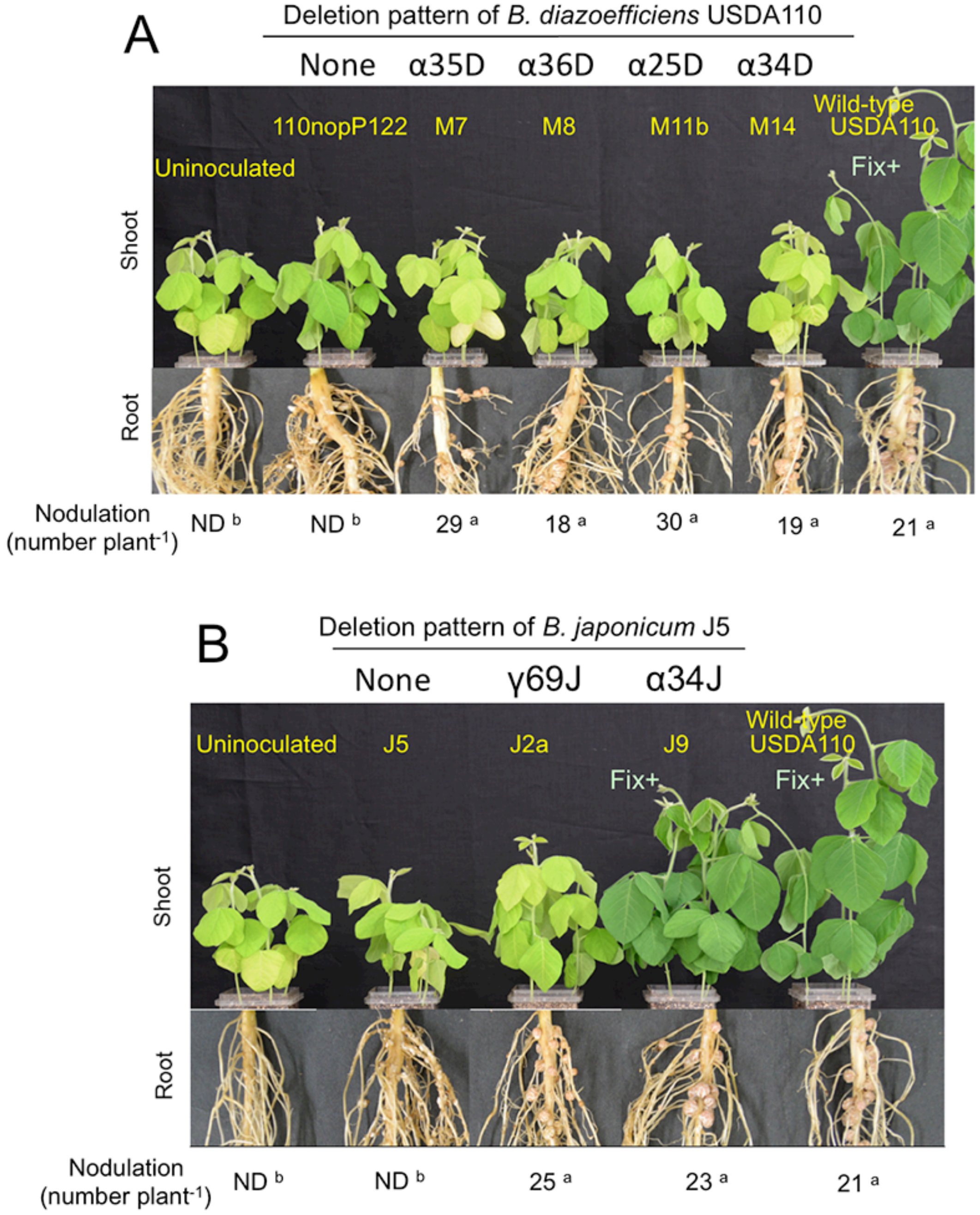

Fig. S3. Symbiotic phenotypes of *Rj2*-soybean plants inoculated with representative derivatives of the different deletion patterns for *Bradyrhizobium diazoefficiens* USDA110 (A) and *B. japonicum* J5 (B). Fix phenotypes of the mutants were examined by comparisons of plant growth between uninoculated controls and wild-type USDA110 inoculations. "Nodulation" is expressed as the average number of nodules per plant (n = 9). Average values with the same letter are not significantly different by Tukey's HSD test ($P < 0.01$). ND means less than 0.34 plant^{-1}.

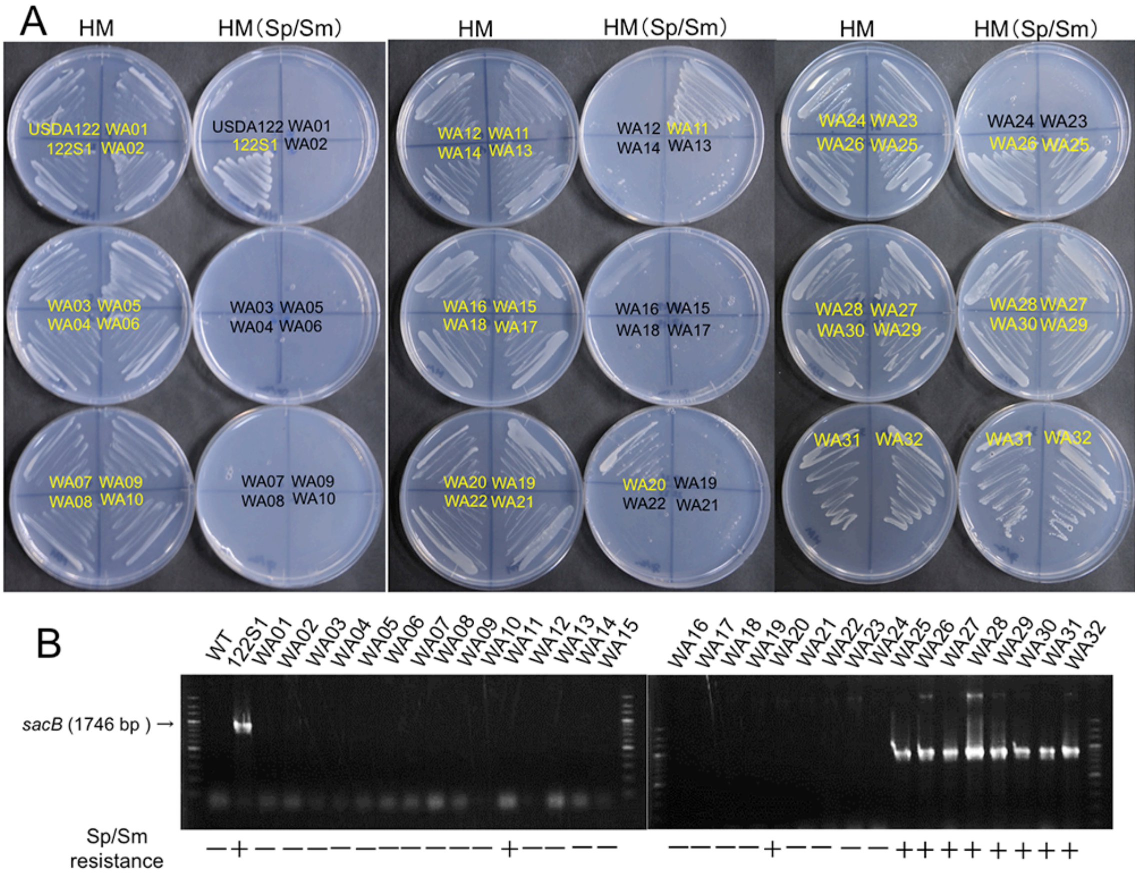

Fig. S4. Antibiotic resistance and *sacB* PCR of 32 sucrose-resistant mutants of *Bradyrhizobium diazefficiens* 122S1. (A) Spectinomycin (Sp) and Streptomycin (Sm) -resistant tests on agar plates. Wild-type USDA122 and 122S1 were sensitive and resistant to Sp/Sm, respectively. Yellow and black letters of the mutant names indicate sensitivity and resistance to Sp/Sm, respectively. (B) PCR analysis of the full-length *sacB* gene (1,786 bp). "+" and "-" indicate resistance and sensitivity to Sp/Sm, respectively. Of the 32 mutants, 22 (WA01~WA10, WA12~WA19, WA21~WA24) were sensitive to Sp/Sm and produced no *sacB* PCR product.

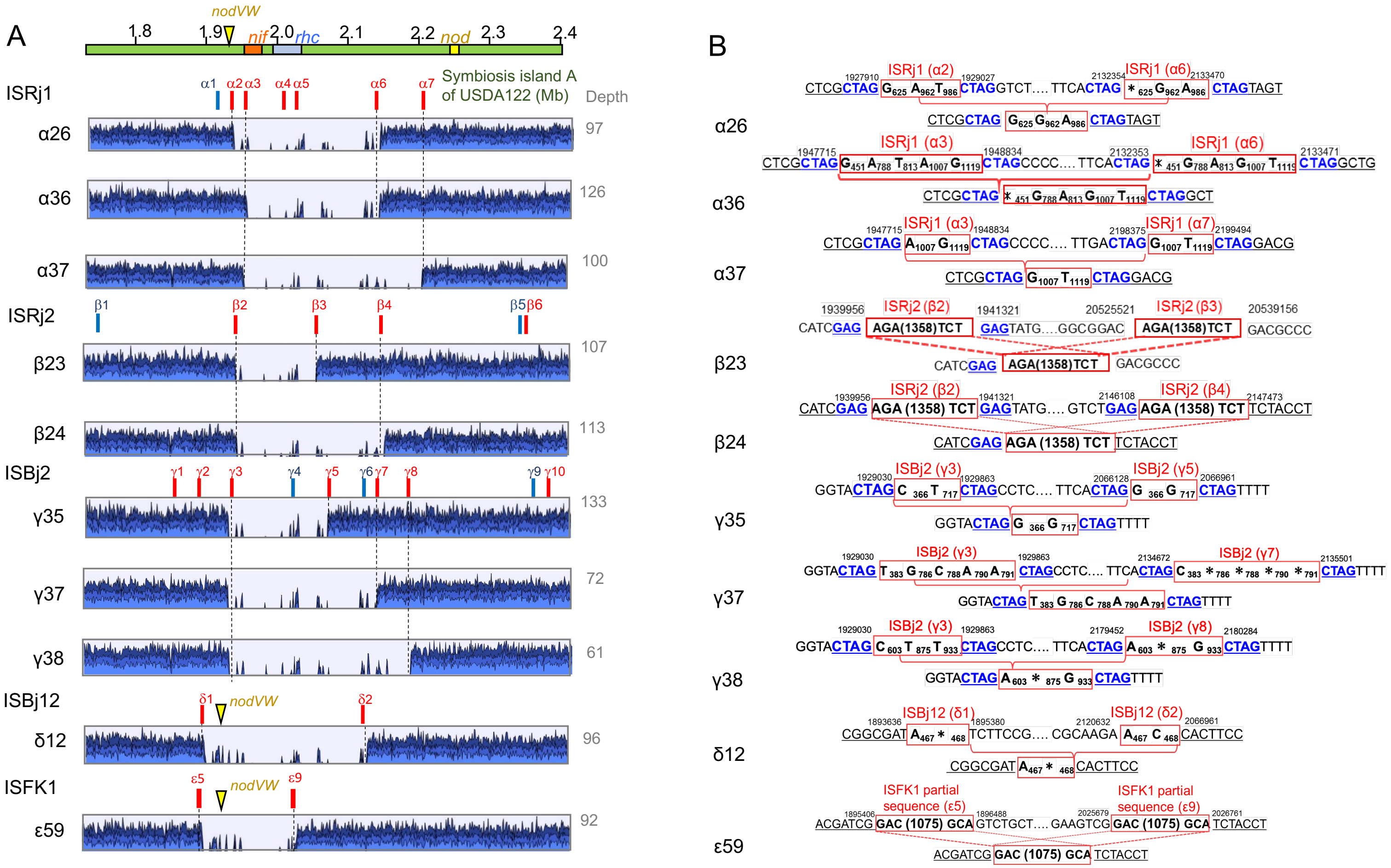

Fig. S5. Mapping profiles of MiSeq reads of 122S1 mutants on the parent genome (A) and their junction sequences (B). Colors and symbols are the same as those in Fig. 2. (A) Representative profiles of α26, α36, α37, β23, β24, γ35, γ37, γ38, δ12, and ε59 were derived from mutants WA03, WA14, WA02, WA22, WA05, WA06, WN25, WN47, WA01, and WA09, respectively (Table S2). (B) Sequence comparisons of the junctions around IS elements between the parent (above) and mutants (below), for respective deletion patterns of *B. diazoefficiens* 122S1.

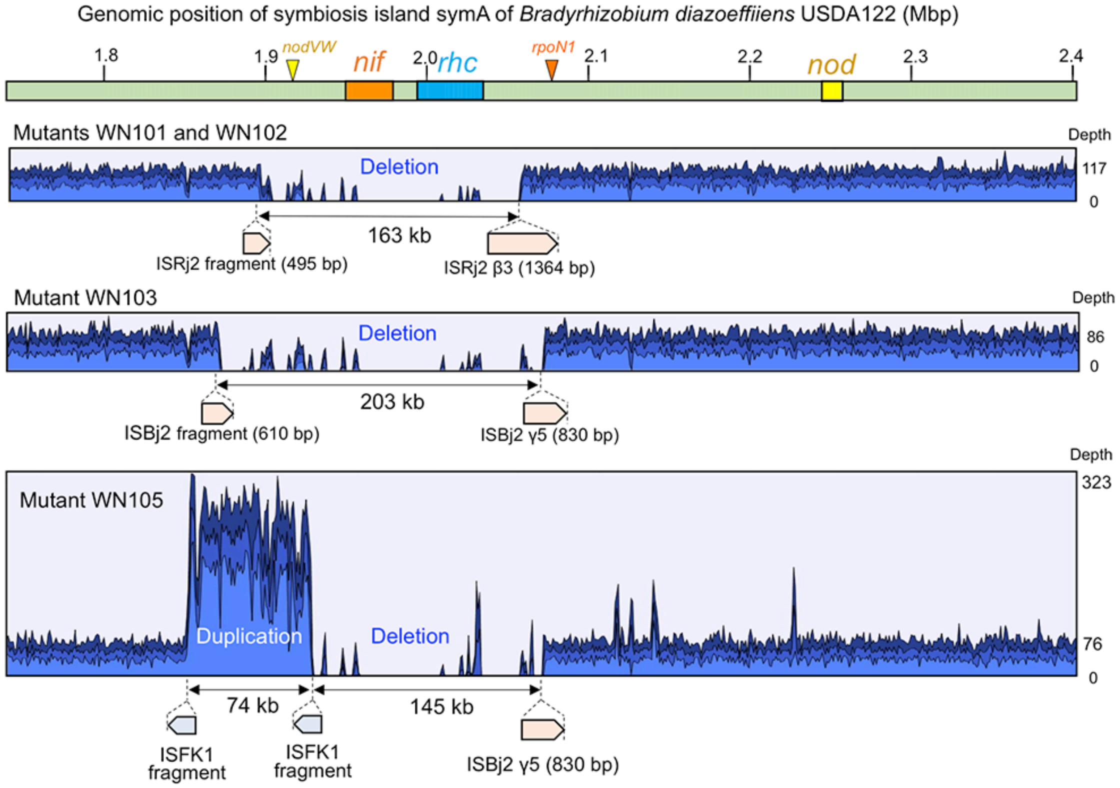

Fig. S6. Mapping profiles of MiSeq reads of mutants WN101, WN102, WN103, and WN105 from 122S1 with *sacB*/*aadA* cassette on symbiosis island of *B. diazozefiiciens* USDA122. The region (163 kb) on the symbiosis island of mutants WN101 and WN102 was deleted between full copy (1364 bp) and a fragment (495 bp) of ISRj2. The region (203 kb) on the symbiosis island of the mutant WN103 was also deleted between full copy (830 bp) and a fragment (610 bp) of ISBj2. The mutant WN105 showed a peculiar profile, including a deletion (145 kb) and duplication (74 kb), where the 74-kb region was likely duplicated approximately four times between two ISFK1 fragments.

A Primer position

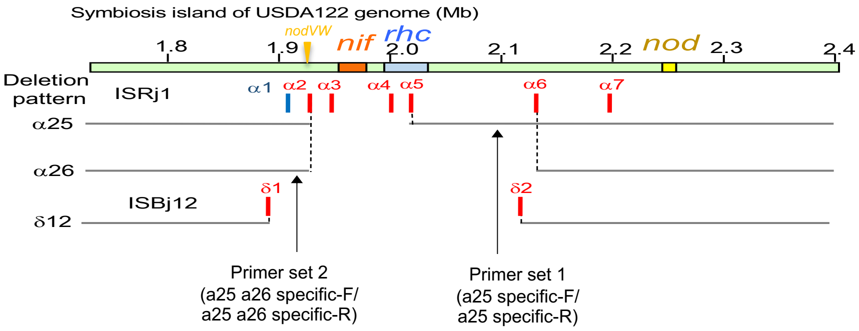

B Multiplex PCR to discriminate mutants by nodule from single inoculation

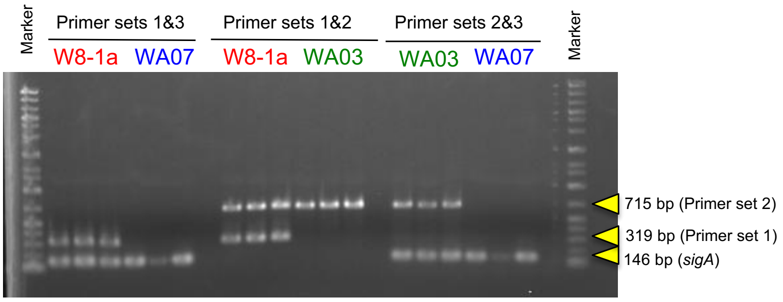

C Nodule number after pairwise mutant inoculation by multiplex PCR

Pairwise Mutants	Replication	W8-1a (α25)	WA07 (δ12)	Total nodule number
W8-1a (α25) vs WA07 (δ12)	1	60	0	60
W8-1a (α25) vs WA07 (δ12)	2	53	0	53
W8-1a (α25) vs WA07 (δ12)	3	40	0	40
Pairwise Mutants	Replication	WA03 (α26)	WA07 (δ12)	Total nodule number
WA03 (α26) vs WA07 (δ12)	1	63	1	64
WA03 (α26) vs WA07 (δ12)	2	46	0	46
WA03 (α26) vs WA07 (δ12)	3	63	1	64
Pairwise Mutants	Replication	W8-1a (α25)	WA03 (α26)	Total nodule number
W8-1a (α25) vs WA03 (α26)	1	60	13	73
W8-1a (α25) vs WA03 (α26)	2	42	7	49
W8-1a (α25) vs WA03 (α26)	3	36	8	44

Fig. S7. Competitive nodulation of pairwise inoculations from deletion mutants W8-1a (α25), WA03 (α26), and WA07 (δ12) by multiplex PCR. (A) Positions of primer set 1 (a25 specific-F/a25 specific-R) and primer set 2 (a25 a26 specific-F/a25 a26 specific-R) on USDA122 symbiosis island, to discriminate the mutants. Primer sets of *sigA* gene on the genome core were used for WA07 (δ12) mutant due to there being no signal primer by sets 1 and 2. These primer sequences are listed in Table S5. (B) Multiplex PCR to discriminate the mutants by nodule lysate from single inoculation experiment. (C) Cell suspensions (1×10^5 cells per mL) were prepared from 5-day-old-cultures of mutant W8-1a, WA03, and WA07. Equal amounts of W8-1a and WA07 cells (1:1) were mixed to evaluate their competitive nodulation. Likewise, other mixed cells (W8-1a & WA03 or WA03 & WA07) were prepared. The pairwise mixed cells were inoculated onto soybean seeds, Hardee (2×10^5 cells per seed). Two plants were cultivated in a Leonard jar assemblage with tree replications for 4 weeks. Nodules were excised from the root systems. Lysate from respective nodules was subjected to the multiplex PCR analysis.

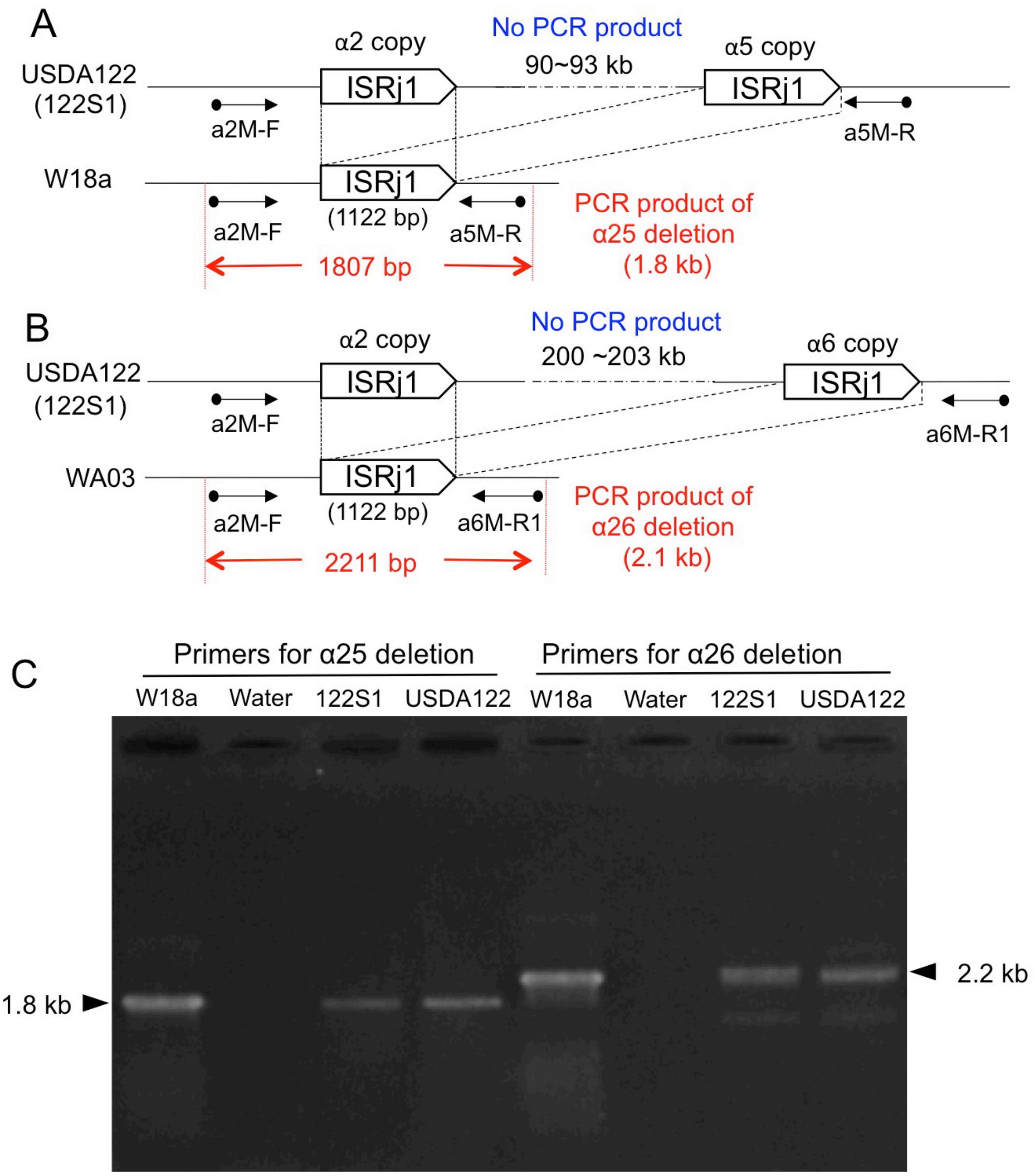

Fig. S8. PCR detection of IS-mediated deletion events in free-living cultures of *Bradyrhizobium diazoefficiens* USDA122 and 122S1. (AB) The existence of ISRj1-mediated deletions during cultivation were examined by PCR reactions of the total DNAs from 5 day-old-cultures of USDA122 and 122S1 for ISRj1-mediated α25 deletion by primers a2M-F and a5M-R (A) and α26 deletion by primers a2M-F and a6M-R1 (B) (Table S3). (C) Agarose gel electrophoresis of PCR products of α25 (A) and α26 deletion events (B) during USDA122 and 122S1 cultivation. Total DNA of mutants W18a (A) and WA03 (B) (Table S2) were used as positive controls, while no template DNA (Water) was used as a negative control. Arrowheads show the expected sizes (1.8 and 2.2 kb) of the PCR products of α25 (A) and α26 deletion events (B) during USDA122 and 122S1 cultivation, respectively.

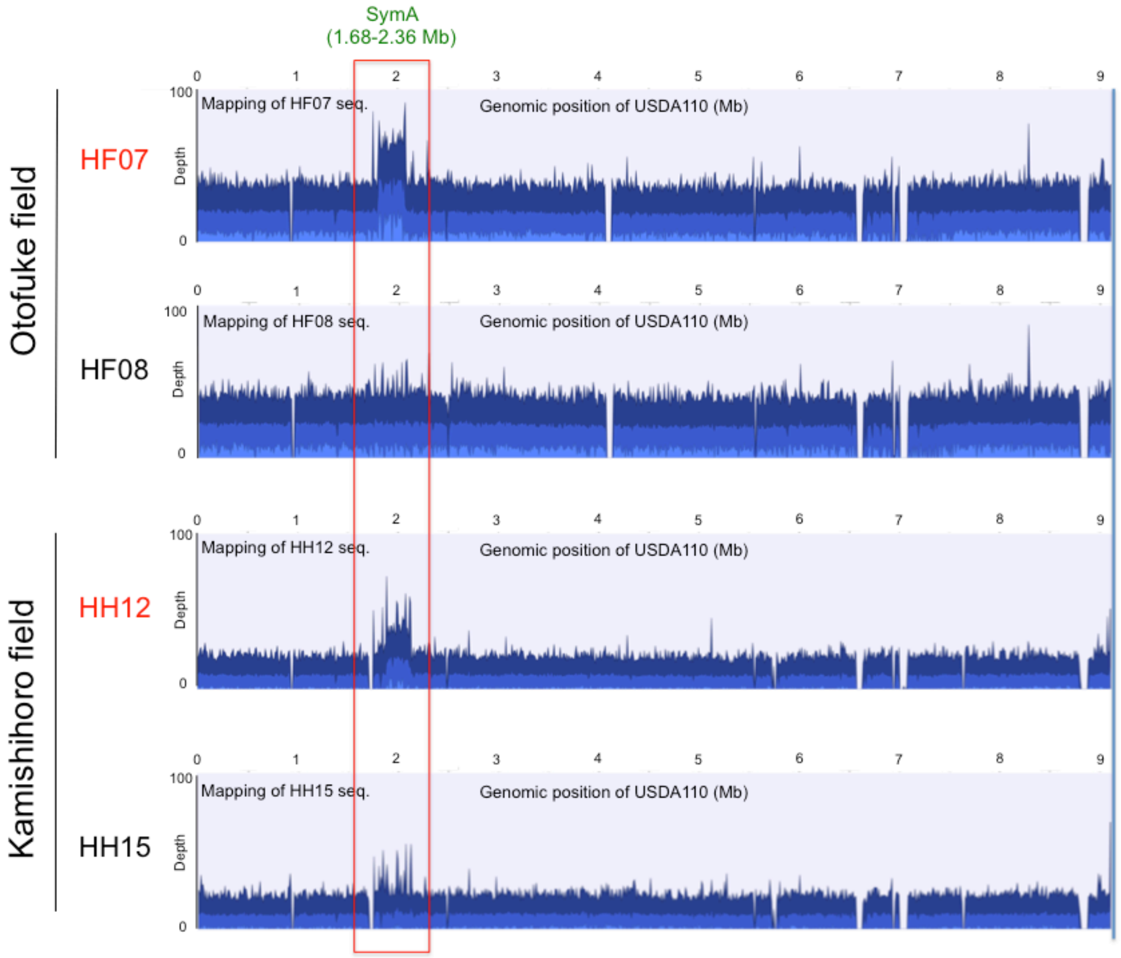

Fig. S9. Mapping profiles of MiSeq reads of *B. diazoefficines* isolates HF07, HF08, HH12, and HH15 on *B. diazoefficines* USDA110^{T} genome. The profile of the HF07 reads showed a heavily mapped region on USDA110 symbiosis island compared with the HF08 profile. The profile of the HH12 reads also showed a heavily mapped region compared with HH15 profile.

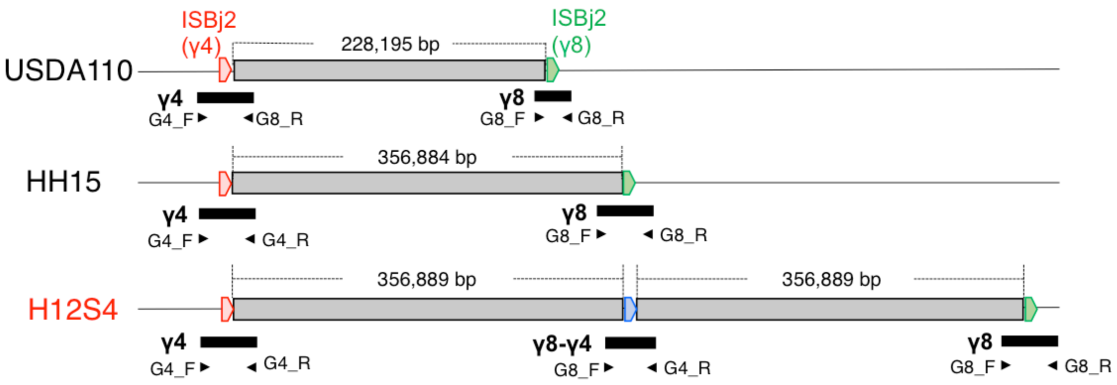

Expected product size (bp)

	Primer sets		
	γ4<br>G4_F<br>G4_R	γ8-γ4<br>G8_F<br>G4_R	γ8<br>G8_F<br>G8_R
USDA 110	2679	No	1495
HH15	2681	No	2946
H12S4	2680	1984	2946

Primer Sequences (5'- 3'):
G4_F: TAGTCCGTCAGTCCCGTCAT
G4_R: GCTCGGTACTTCATCCTCCG
G8_F: CCGATACACATGCCGGATCA
G8_R: GATGGAGATCCTCAAGCGGG

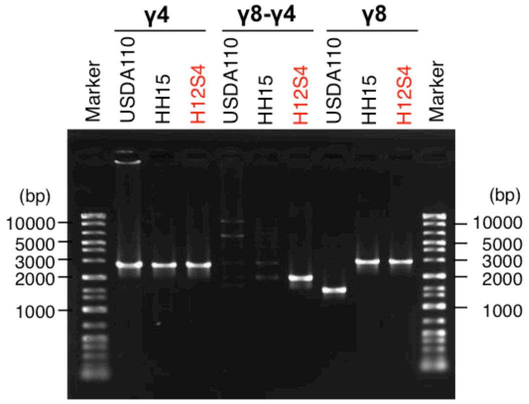

Fig. S10. Verification of tandem duplication structure in symbiosis island of *B. diazoefficiens* H12S4 from Otofuke field by PCR analysis. All PCR products were sequenced to verify the structures of the tandem duplication in H12S4 and the non-duplication in HH15, resulting in complete genomes of H12S4 (AP022639) and HH15 (AP022641).

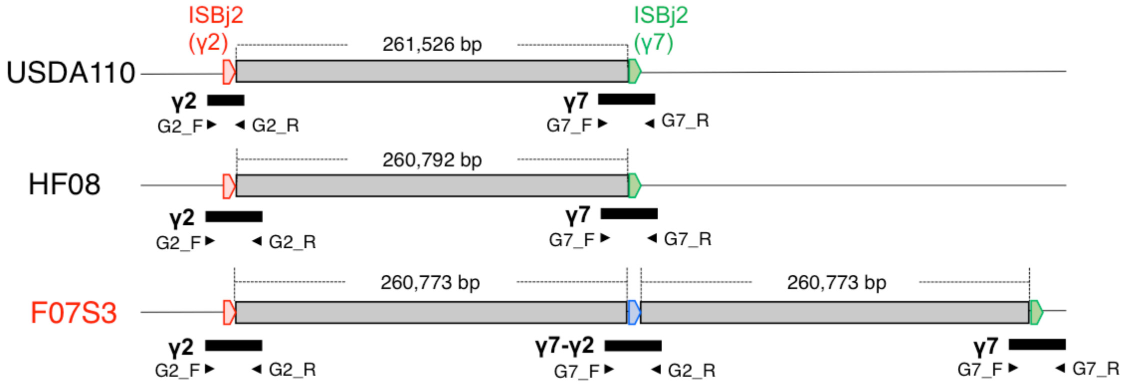

Expected product size (bp)

	Primer sets		
	γ2<br>G2_F<br>G2_R	γ7-γ2<br>G7_F<br>G2_R	γ7<br>G7_F<br>G7_R
USDA 110	1685	No	4714
HF08	3598	No	4717
F07S3	3609	3632	4717

Primer Sequences (5'- 3'):
G2_F: TTGGCTAACACGCTCGAACT
G2_R: TATCTCGCCAATCTTCCGGC
G7_F: CTTTGTTCAACAAGCGGCGA
G7_R: GCATCGCACCTAGCACTACT

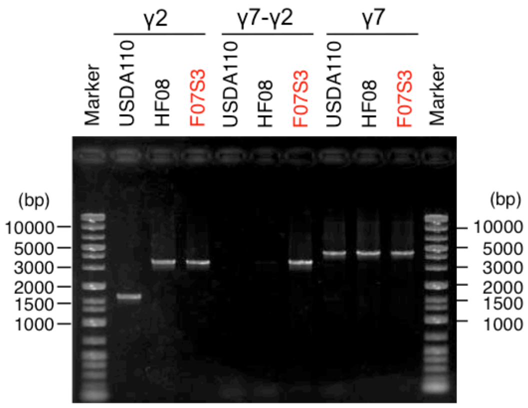

Fig. S11. Verification of tandem duplication structure in symbiosis island of *B. diazoefficiens* F07S3 from Kamishihoro field by PCR analysis. All PCR products were sequenced to verify the structures of the tandem duplication in F07S3 and the non-duplication in HF08, resulting in complete genomes of F07S3 (AP022638) and HF08 (AP022640).

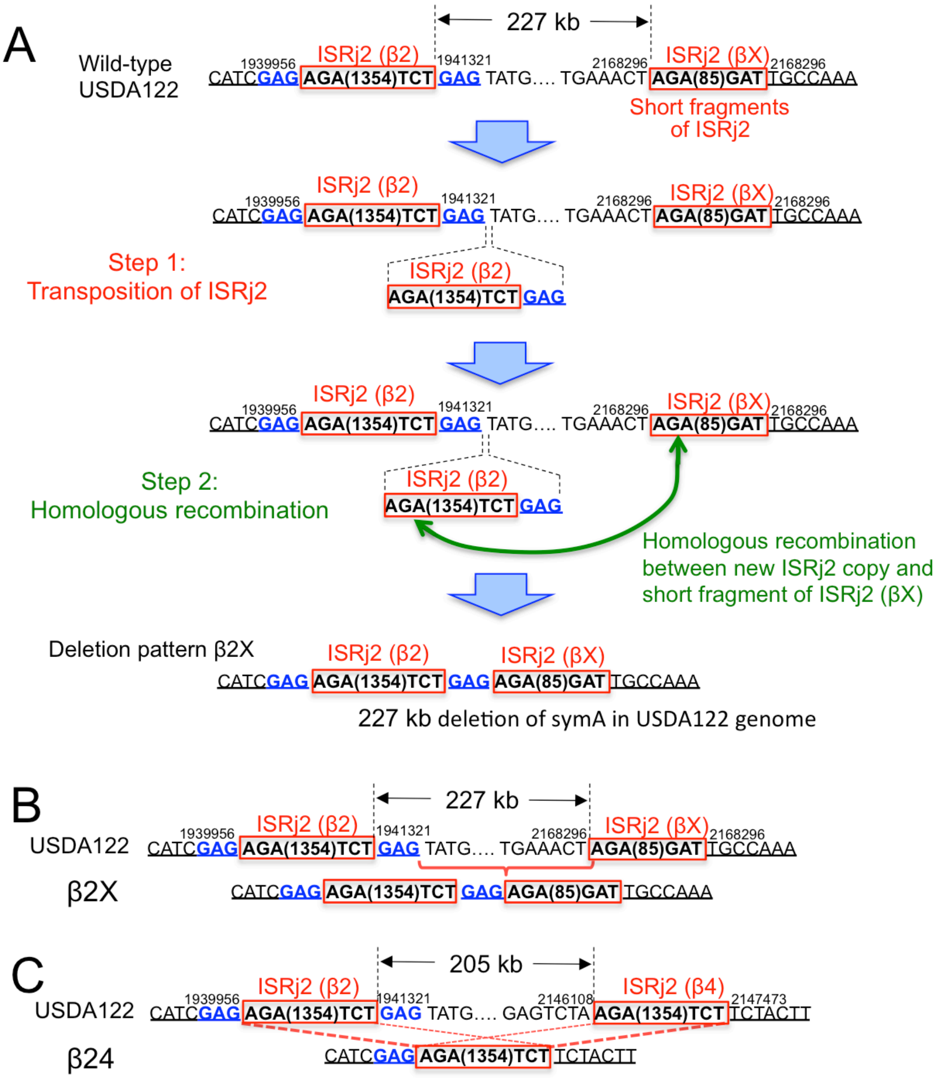

Fig. S12. Estimation of generation steps of the mutants having deletion pattern β2X in symbiosis island A from USDA122 genome (A), based on the sequence comparison at the junctions of IS elements between wild-type parent and two mutants with deletion pattern β2X (B) and β24 (C) (Fig. 2). The mutants having deletion pattern β2X were likely generated through two steps including (i) transposition (insertion) of ISRj2 and (ii) homologous recombination. Note that the deletion patter β24 (C) was simply generated by homologous recombination that differed from the deletion pattern β2X (B). Red box and blue letter indicate IS elements and putative target duplicates, respectively. Upper figures on DNA sequence show genomic position on USDA122 genomes (Sugawara et al. 2017).

Table S1. List of IS elements on the genomes of USDA122, USDA110, and J5

Strain	IS	Position	Symbiosis island[a]	Length (bp)	Direction[b]	Start	End
USDA122	ISRj1	α1	A	1116	<<	1907569	1906454
USDA122	ISRj1	α2	A	1116	>>	1927911	1929026
USDA122	ISRj1	α3	A	1116	>>	1947717	1948832
USDA122	ISRj1	α4	A	1116	>>	2002304	2003419
USDA122	ISRj1	α5	A	1116	>>	2018932	2020047
USDA122	ISRj1	α6	A	1115	>>	2132355	2133469
USDA122	ISRj1	α7	A	1116	>>	2198377	2199492
USDA122	ISRj1	-	-	1116	>>	7156235	7157350
USDA122	ISRj1	-	-	1116	<<	8859054	8857939
USDA122	ISRj1	-	C	1114	>>	9085500	9086613
USDA122	ISRj2	-	C	1364	>>	14651	16014
USDA122	ISRj2	β1	A	1364	<<	1743042	1741679
USDA122	ISRj2	β2	A	1364	>>	1939957	1941320
USDA122	ISRj2	β3	A	1364	>>	2052552	2053915
USDA122	ISRj2	β4	A	1364	>>	2146109	2147472
USDA122	ISRj2	β5	A	1362	<<	2344302	2342941
USDA122	ISRj2	β6	A	1364	>>	2352835	2354198
USDA122	ISRj2	-	-	1364	<<	7878835	7877472
USDA122	ISRj2	-	C	1364	>>	9087818	9089181
USDA122	ISRj2	-	C	1364	>>	9108784	9110147
USDA122	ISFK1	-	-	2592	>>	9074536	9077127
USDA122	ISBj6_B	-	A	1431	<<	1851968	1850538
USDA122	ISBj6_B	-	A	1437	>>	2137428	2138864
USDA122	ISB27	-	A	2441	<<	1911463	1909023
USDA122	ISBj2	γ1	A	828	>>	1847778	1848605
USDA122	ISBj2	γ2	A	828	>>	1882860	1883687
USDA122	ISBj2	γ3	A	832	>>	1929031	1929862
USDA122	ISBj2	γ4	A	832	<<	2015464	2014633
USDA122	ISBj2	γ5	A	832	>>	2066129	2066960
USDA122	ISBj2	γ6	A	832	<<	2115967	2115136
USDA122	ISBj2	γ7	A	828	>>	2134673	2135500
USDA122	ISBj2	γ8	A	831	>>	2179453	2180283
USDA122	ISBj2	γ9	A	832	<<	2356443	2355612
USDA122	ISBj2	γ10	A	832	>>	2377430	2378261
USDA122	ISBj2	-	-	832	<<	7402797	7401966
USDA122	ISBj3	-	A	1284	>>	1733268	1734551
USDA122	ISBj4	-	-	1576	<<	3341304	3339729
USDA122	ISBj4	-	-	1576	<<	7244643	7243068
USDA122	ISBj4	-	-	1575	>>	7866060	7867634
USDA122	ISBj4	-	-	1574	>>	7871634	7873207
USDA122	ISBj5	-	C	1185	>>	21908	23092
USDA122	ISBj5	-	C	1185	>>	9061139	9062323
USDA122	ISBj5	-	-	1185	>>	7906286	7907470
USDA122	ISBj7	-	A	813	>>	1856539	1857351
USDA122	ISBj7	-	A	822	<<	1878867	1878046
USDA122	ISBj7	-	A	816	<<	1885563	1884748
USDA122	ISBj8	-	-	1622	>>	221068	222689
USDA122	ISBj8	-	A	1622	<<	2292287	2290666
USDA122	ISBj8	-	A	1622	<<	2340771	2339150

USDA122	ISBj8	-	-	1622	>>	3993693	3995314
USDA122	ISBj8	-	-	1622	<<	3998574	3996953
USDA122	ISBj8	-	-	1621	<<	4065949	4064329
USDA122	ISBj8	-	-	1622	<<	4105281	4103660
USDA122	ISBj8	-	-	1622	>>	5243253	5244874
USDA122	ISBj8	-	-	1622	>>	7296012	7297633
USDA122	ISBj8	-	-	1622	<<	8361034	8359413
USDA122	ISBj10	-	A	2298	<<	2170684	2168387
USDA122	ISBj11	-	A	2648	>>	2222462	2225109
USDA122	ISBj12	δ1	A	1743	>>	1893637	1895379
USDA122	ISBj12	δ2	A	1744	>>	2120633	2122376
USDA122	ISBj12	-	C	1743	<<	9131280	9129538
USDA122	ISBj2_B	-	A	1509	<<	2024114	2022606
USDA122	ISBj2_B	-	A	1507	<<	2195668	2194162
USDA110	ISRj1	α1D	A	1116	>>	1860456	1861571
USDA110	ISRj1	α2D	A	1116	>>	1883172	1884287
USDA110	ISRj1	α3D	A	1116	>>	1904907	1906022
USDA110	ISRj1	α4D	A	1116	>>	1962375	1963490
USDA110	ISRj1	α5D	A	1116	>>	1979002	1980117
USDA110	ISRj1	α6D	A	1116	>>	2066180	2067295
USDA110	ISRj1	α7D	A	1116	>>	2244775	2245890
USDA110	ISRj1	α8D	A	1017	>>	2338198	2339214
USDA110	ISRj1	-	-	1116	<<	4667558	4666443
USDA110	ISRj1	-	-	1116	<<	5142513	5141398
USDA110	ISRj1	-	-	1116	>>	5397253	5398368
USDA110	ISRj1	-	-	1117	<<	6290952	6289836
USDA110	ISRj1	-	-	1116	>>	8285049	8286164
USDA110	ISRj1	-	C	1114	>>	9024824	9025937
USDA110	ISRj1	-	C	1116	<<	9080899	9079784
USDA110	ISRj2	-	C	1364	>>	14651	16014
USDA110	ISRj2	β1D	A	1364	<<	1766041	1764678
USDA110	ISRj2	β2D	A	1364	>>	1897147	1898510
USDA110	ISRj2	β3D	A	1364	>>	2079936	2081299
USDA110	ISRj2	β4D	A	1364	<<	2294303	2292940
USDA110	ISRj2	-	-	1364	<<	8331567	8330204
USDA110	ISRj2	-	C	1364	>>	9027142	9028505
USDA110	ISRj2	-	C	1364	>>	9050401	9051764
USDA110	ISFK1	-	C	2592	>>	9013859	9016450
USDA110	ISBj6_B	-	A	1447	<<	1808423	1806977
USDA110	ISBj6_B	-	A	1437	>>	2071254	2072690
USDA110	ISB27	-	A	2440	>>	1856563	1859002
USDA110	ISBj2	γ1D	A	832	<<	1755653	1754822
USDA110	ISBj2	γ2D	A	830	>>	1806143	1806972
USDA110	ISBj2	γ3D	A	832	>>	1839309	1840140
USDA110	ISBj2	γ4D	A	830	>>	1884292	1885121
USDA110	ISBj2	γ5D	A	831	<<	1975534	1974704
USDA110	ISBj2	γ6D	A	832	>>	2027184	2028015
USDA110	ISBj2	γ7D	A	828	>>	2068499	2069326
USDA110	ISBj2	γ8D	A	831	>>	2113280	2114110
USDA110	ISBj2	-	-	832	>>	4286526	4287357
USDA110	ISBj3	-	A	1284	>>	1683969	1685252
USDA110	ISBj3	-	-	1284	>>	4320931	4322214
USDA110	ISBj4	-	-	1576	<<	5124491	5122916
USDA110	ISBj4	-	-	1576	>>	5747867	5749442

USDA110	ISBj4	-	-	1578	>>	6668587	6670164
USDA110	ISBj4	-	-	1576	<<	8290727	8289152
USDA110	ISBj4	-	-	1576	>>	8460058	8461633
USDA110	ISBj4	-	C	1578	<<	8988744	8987167
USDA110	ISBj5	-	C	1185	>>	21908	23092
USDA110	ISBj5	-	-	1185	>>	2702539	2703723
USDA110	ISBj5	-	-	1185	>>	8293172	8294356
USDA110	ISBj5	-	C	1185	>>	8985098	8986282
USDA110	ISBj6	-	C	1656	>>	9090881	9092536
USDA110	ISBj7	-	A	814	>>	1812994	1813807
USDA110	ISBj7	-	A	824	<<	1835316	1834493
USDA110	ISBj7	-	A	816	<<	1842016	1841201
USDA110	ISBj9	-	-	1454	<<	5742278	5740825
USDA110	ISBj9	-	C	1454	>>	9058516	9059969
USDA110	ISBj10	-	A	2298	<<	2104511	2102214
USDA110	ISBj11	-	A	2649	>>	1870866	1873514
USDA110	ISBj11	-	A	2649	<<	2157908	2155260
USDA110	ISBj11	-	-	2648	<<	3893693	3891046
USDA110	ISBj11	-	-	2649	<<	5545696	5543048
USDA110	ISBj12	δ1D	A	1743	<<	2053699	2051957
USDA110	ISBj2_B	-	A	1509	<<	1984184	1982676
USDA110	ISBj2_B	-	A	1507	<<	2133262	2131756
J5	ISRj1	-	-	1116	>>	4759876	4760991
J5	ISRj1	α1J	A	1106	<<	8749109	8748004
J5	ISRj1	α2J	A	1116	>>	8896185	8897300
J5	ISRj1	α3J	A	1116	>>	9213436	9214551
J5	ISRj1	α4J	A	1116	>>	9274269	9275384
J5	ISRj1	α5J	A	1116	<<	9329973	9328858
J5	ISRj1	α6J	A	1116	<<	9370647	9369532
J5	ISRj1	-	C	1114	>>	10053968	10055081
J5	ISRj2	-	C	1364	>>	10056286	10057649
J5	ISRj2	-	C	1364	>>	10079545	10080908
J5	ISRj2	-	C	1364	>>	14651	16014
J5	ISFK1	-	C	2592	>>	10043003	10045594
J5	ISBj6_B	-	A	1447	>>	9421611	9423057
J5	ISB27	-	A	2441	<<	9374541	9372101
J5	ISBj2	-	-	832	>>	3153883	3154714
J5	ISBj2	γ1J	A	832	>>	8584626	8585457
J5	ISBj2	γ2J	A	832	<<	8746800	8745969
J5	ISBj2	γ3J	A	831	>>	8763636	8764466
J5	ISBj2	γ4J	A	832	>>	8775461	8776292
J5	ISBj2	γ5J	A	832	>>	8779411	8780242
J5	ISBj2	γ6J	A	832	<<	8809961	8809130
J5	ISBj2	γ7J	A	832	>>	8820377	8821208
J5	ISBj2	γ8J	A	831	>>	9262225	9263055
J5	ISBj2	γ9J	A	832	<<	9350631	9349800
J5	ISBj2	γ10J	A	832	>>	9376274	9377105
J5	ISBj2	γ11J	A	832	<<	9392627	9391796
J5	ISBj2	-	C	832	>>	10006560	10007391
J5	ISBj3	-	A	1280	<<	8880495	8879216
J5	ISBj4	-	C	1578	<<	10011041	10009464
J5	ISBj5	-	C	1185	>>	21908	23092
J5	ISBj5	-	C	1185	>>	10007395	10008579
J5	ISBj6	-	C	1656	>>	10117446	10119101

J5	ISBj7	-	A	814	<<	9417040	9416227
J5	ISBj7	-	A	823	>>	9396619	9397441
J5	ISBj7	-	A	822	>>	9401027	9401848
J5	ISBj7	-	A	816	>>	9389920	9390735
J5	ISBj11	-	-	2649	>>	3343642	3346290
J5	ISBj11	-	A	2649	>>	8928892	8931540
J5	ISBj11	-	A	2649	>>	9215237	9217885
J5	ISBj12	-	C	1743	<<	10133395	10131653
J5	ISBj2_B	-	A	1509	>>	9255715	9257223

[a] Symbiosis island C was previously defined by Kaneko *et al.* (10) but was only recently regarded as a genomic island (personal communication).

[b] “>>” and “<<” indicate clockwise and anticlockwise directions on the genomes, respectively.

Table S2. List of the mutants having partial deletions of symbiosis islands [a]

Mutant	Background	PCR analysis			Deletion pattern	Origin	Accession number	
		nifH	*rhcJ*	*nodC*			MiSeq read	Junction seq.
W18a	USDA122	-	-	+	α25	Exp. 1		
W20	USDA122	-	-	+	β2X	Exp. 1	DRX161341	LC471411
W22	USDA122	-	-	+	α25	Exp. 1		
W23	USDA122	-	-	+	β23	Exp. 1		
W24a	USDA122	-	-	+	α25	Exp. 1		
W24b	USDA122	-	-	+	α25	Exp. 1		
W25	USDA122	-	-	+	α36	Exp. 1	DRX161325	LC471412
W27	USDA122	+	-	+	α45	Exp. 1	DRX161334	LC471413
W29	USDA122	-	-	+	α37	Exp. 1	DRX161333	LC471414
W30	USDA122	-	-	+	α36	Exp. 1	DRX161326	LC471415
W33a	USDA122	-	-	+	α25	Exp. 1		
W33b	USDA122	-	-	+	α36	Exp. 1	DRX161327	LC471416
W35	USDA122	-	-	+	β24	Exp. 1	DRX161339	LC471417
W36	USDA122	-	-	+	α27	Exp. 1		
W37a	USDA122	+	-	+	α45	Exp. 1	DRX161335	LC471418
W39	USDA122	-	-	+	α25	Exp. 1		
W40	USDA122	-	-	+	α25	Exp. 1		
W42a	USDA122	-	-	+	α36	Exp. 1	DRX161328	LC471419
W42b	USDA122	-	-	+	α36	Exp. 1	DRX161329	LC471420
W43a	USDA122	-	-	+	α36	Exp. 1	DRX161330	LC471421
W43b	USDA122	-	-	+	α36	Exp. 1	DRX161331	LC471422
W43c	USDA122	-	-	+	α36	Exp. 1	DRX161332	LC471423
W44	USDA122	+	-	+	α45	Exp. 1	DRX161336	LC471424
W46a	USDA122	-	-	+	β24	Exp. 1		LC471425
W46b	USDA122	+	-	+	α46	Exp. 1	DRX161337	LC471426
W48	USDA122	-	-	+	α25	Exp. 1		
W49	USDA122	-	-	+	β24	Exp. 1	DRX161340	LC471427
W13	USDA122	-	-	+	α27	Exp. 2		
W14b	USDA122	-	-	+	α25	Exp. 2		
W17	USDA122	-	-	+	α36	Exp. 2	DRX161324	LC471410
HG12[b]	122GFP	+	+	+	α57	Exp. 3	DRX161338	LC471428
WA01	122S1	-	-	+	δ12	Exp. 4	DRX161351	LC471437
WA02	122S1	-	-	+	α37	Exp. 4	DRX161352	LC471438
WA03	122S1	-	-	+	α26	Exp. 4	DRX161353	LC471439
WA04	122S1	-	-	+	δ12	Exp. 4	DRX161354	LC471440
WA05	122S1	-	-	+	β24	Exp. 4	DRX161355	LC471441
WA06	122S1	-	-	+	γ35	Exp. 4	DRX161356	LC471442
WA07	122S1	-	-	+	δ12	Exp. 4	DRX161357	LC471443
WA08	122S1	+	+	+	εX9	Exp. 4	DRX161358	LC471444
WA09	122S1	-	-	+	ε59	Exp. 4	DRX161359	LC471445
WA10	122S1	-	-	+	δ12	Exp. 4	DRX161360	LC471446
WA12	122S1	-	-	+	α37	Exp. 4	DRX170987	LC494630
WA13	122S1	-	-	+	β24	Exp. 4	DRX170988	LC494632
WA14	122S1	-	-	+	α36	Exp. 4	DRX170989	LC494629
WA15	122S1	-	-	+	γ35	Exp. 4	DRX170990	LC494634

WA16	122S1	-	-	+	β24	Exp. 4	DRX170991	LC494633
WA17	122S1	-	-	+	δ12	Exp. 4	DRX170992	LC494623
WA18	122S1	-	-	+	δ12	Exp. 4	DRX170993	LC494624
WA19	122S1	-	-	+	δ12	Exp. 4	DRX170994	LC494625
WA21	122S1	-	-	+	δ12	Exp. 4	DRX170995	LC494626
WA22	122S1	-	-	+	β23	Exp. 4	DRX170996	LC494631
WA23	122S1	-	-	+	δ12	Exp. 4	DRX170997	LC494627
WA24	122S1	-	-	+	δ12	Exp. 4	DRX170998	LC494628
WN01	122S1				α45	Exp. 5		
WN02	122S1				γ37	Exp. 5	DRX223741	LC552942
WN03	122S1				γ35	Exp. 5		
WN04	122S1				β24	Exp. 5		
WN05	122S1				α36	Exp. 5		
WN06	122S1				δ12	Exp. 5		
WN07	122S1				δ12	Exp. 5		
WN08	122S1				δ12	Exp. 5		
WN09	122S1				γ35	Exp. 5		
WN10	122S1				α25	Exp. 5		
WN11	122S1				α26	Exp. 5		
WN12	122S1				δ12	Exp. 5		
WN13	122S1				δ12	Exp. 5		
WN14	122S1				β24	Exp. 5		
WN15	122S1				α26	Exp. 5		
WN16	122S1				α26	Exp. 5		
WN17	122S1				α26	Exp. 5		
WN18	122S1				α26	Exp. 5		
WN19	122S1				α26	Exp. 5		
WN20	122S1				δ12	Exp. 5		
WN21	122S1				β23	Exp. 5		
WN22	122S1				α36	Exp. 5		
WN23	122S1				β24	Exp. 5		
WN24	122S1				δ12	Exp. 5		
WN25	122S1				γ37	Exp. 5	DRX223742	LC552943
WN26	122S1				α27	Exp. 5		
WN27	122S1				δ12	Exp. 5		
WN28	122S1				α25	Exp. 5		
WN29	122S1				α37	Exp. 5		
WN30	122S1				δ12	Exp. 5		
WN31	122S1				δ12	Exp. 5		
WN32	122S1				δ12	Exp. 5		
WN33	122S1				δ12	Exp. 5		
WN34	122S1				α26	Exp. 5		
WN35	122S1				α26	Exp. 5		
WN36	122S1				δ12	Exp. 5		
WN37	122S1				δ12	Exp. 5		
WN38	122S1				α27	Exp. 5		
WN39	122S1				α26	Exp. 5		
WN40	122S1				α36	Exp. 5		
WN41	122S1				γ35	Exp. 5		
WN42	122S1				α25	Exp. 5		

WN43	122S1				β23	Exp. 5		
WN44	122S1				α25	Exp. 5		
WN45	122S1				δ12	Exp. 5		
WN46	122S1				α25	Exp. 5		
WN47	122S1				γ38	Exp. 5	DRX223743	LC552945
WN48	122S1				α26	Exp. 5		
WN49	122S1				α27	Exp. 5		
WN50	122S1				γ37	Exp. 5	DRX223744	LC552944
WN51	122S1				β24	Exp. 5		
WN52	122S1				β23	Exp. 5		
WN53	122S1				α37	Exp. 5		
WN101	122S1				ISRj2 fragment	Exp. 5	DRX225175	
WN102	122S1				ISRj2 fragment	Exp. 5	DRX225176	
WN103	122S1				ISBj2 fragment	Exp. 5	DRX225177	
WN104[c]	122S1				Group 2 intron	Exp. 5	DRX225178	
WN105	122S1				Duplication and deletion	Exp. 5	DRX223740	
WN106[d]	122S1				Non-homologous	Exp. 5	DRX225179	
W3-2a	USDA122	-	-	+	α25	Exp. 1	DRX086801	LC471448
W8-1a	USDA122	-	-	+	α25	Exp. 1	DRX086802	LC471449
W9-3a	USDA122	-	-	+	α25	Exp. 1	DRX086806	LC471450
W8-1b	USDA122	-	-	+	α27	Exp. 1	DRX086803	LC471451
W1-1a	USDA122	-	-	+	β23	Exp. 1	DRX086799	LC471447
M14	110*nopP*$_{122}$	-	+	+	α34D	Exp. 1	DRX161342	LC471433
M7	110*nopP*$_{122}$	-	-	+	α35D	Exp. 1	DRX161343	LC471429
M8	110*nopP*$_{122}$	-	-	+	α36D	Exp. 1	DRX161344	LC471430
M11b	110*nopP*$_{122}$	-	-	+	α25D	Exp. 1	DRX161345	LC471431
M11c	110*nopP*$_{122}$	-	-	+	α25D	Exp. 1	DRX161346	LC471432
J9	J5	-	+	+	α34J	Exp. 1	DRX161347	LC471435
J10	J5	-	+	+	α34J	Exp. 1	DRX161348	LC471436
J2a	J5	-	-	+	γ69J	Exp. 1	DRX161349	LC471434

[a] Mutants from *B. diazoefficiens* USDA122, *B. diazoefficiens* 110*nopP*$_{122}$, and *B. japonicum* J5 were obtained from three-independent experiments by *Rj2*-system (Exp. 1 ~ Exp. 3). Isolates with the prefixes WA and WN were obtained from 122S1 during free-living growth by the *sacB* system (Exp. 4 and Exp. 5) (see text). Blank in "Accession number" indicates that the deletion patterns were verified by PCR analysis. The mutants of experiment 5 (Exp. 5: 2nd *sacB* experiment) were not subjected to PCR analysis for *nifH*, *rhcJ,* and *nodC* genes

[b] Mutant HG12 was obtained from nodules of *Rj2*-soybean cv. Hardee, inoculated with 122GFP (Table S4), which is GFP-tagged USDA122 using pRJPaph-bjGFP (12).

[c] Partial genome deletion was occurred via group 2 introns on mutant WN104.

[d] Mutant WN106 showed non-homologous deletion pattern.

Table S3. Bacterial strains and plasmids used in this study [a]

Strain or plasmid	Relevant characteristic(s)	Reference or source
Bradyrhizobium diazoefficiens strains		
USDA110	Wild type, compatible strain with *Rj2* soybean, Px^r	USDA, Beltsville
USDA122	Wild type, incompatible strain with *Rj2* soybean, Px^r	13
110*nopP*$_{122}$	USDA 110 exchanged *nopP* to USDA122-type gene, Px^r	6
122GFP	GFP-labeled USDA122 by pRJPaph-bjGFP, Px^r, Tc^r	This study
122S1	USDA122 inserted *sacB* and Ω casette between ISRj1α4 and α5, Suc^r, Sp^r, Sm^r	This study
Bradyrhizobium japonicum strain		
J5	Wild type, incompatible strain with *Rj2* soybean, Px^r	7
Escherichia coli strains		
DH5α	F−, Φ80d *lacZ*ΔM15, Δ(l*acZYA*-*argF*)U169, *deoR, recA1, endA1*, *hsdR17*(rK- mK+), *phoA*, *supE44*, λ−, *thi*-1, *gyrA96*, *relA1*	Nippon Gene
Plasmids		
pK18*mob*	Mobilizable vector with pMB1 replicon, Km^r	14
pK18*mobsacB*	pK18mob derivative with *sacB*, Km^r	14
pHP45Ω	plasmid carrying Ωcassette ; Sm^r, Sp^r	15
pRK2013	ColE1 replicon carrying RK2 transfer genes, Km^r	16
pRJPaph-bjGFP	Paph-*bjGFP* for integration downstream of ScoI, Tc^r	12

[a] Km^r, kanamycin resistant; Px^r, polymyxin B resistant; Sp^r, spectinomycin resistant, Sm^r, streptomycin resistant.

Table S4. Polymorphisms of *sacB*/SacB in 8 mutants showing positive *sacB* PCR products and antibiotic resistance to spectinomycin (Sp) and streptomycin (Sm).

Mutants	Change of sequence of *sacB*/SacB	
	Nucleotide	Amino acid
WA25	c.930_delA	p.Asn310fs
WA26	c.681_682insA	p.Asn227fs
WA27	c.180_181insA	p.Asn60fs
WA28	c.966_967insA	p.Tyr322fs
WA29	c.180_181insA	p.Asn60fs
WA30	c.105_106insA, c.141A>C	p.Pro35fs
WA31	c.297_298insA	p.Asn99fs
WA32	c.32A>C, c.957_958insA	p.Thr11Pro, p.Asn319fs

Table S5. Oligonucleotide primers used for the deletion analyses.

Name	Sequence (5' - 3')	Target gene of position	Purpose
nifH-f	CTCGACTCGCCTTATTCTGC	*nifH* in USDA122, internal region	Examination of *nifH* region
nifH-r	TAGATCTCCTGCGCCTTGTT	*nifH* in USDA122, internal region	Examination of *nifH* region
rhcJ-f	TTCGCGTGTTTCTTGCTATG	*rhcJ* in USDA122, internal region	Examination of *rhcJ* region
rhcJ-r	AGCCATGTCGAATGAAGACC	*rhcJ* in USDA122, internal region	Examination of *rhcJ* region
nodC-f	CTCCTCGCCATTTCATCACT	*nodC* in USDA122, internal region	Examination of *nodC* region
nodC-r	CGCCTTACGAATAGGAGCAG	*nodC* in USDA122, internal region	Examination of *nodC* region
T1 F-F	AGTGAATTCCCGAATCAACACGTAGGCAA	Intergenetic region (2015413-2016412) with EcoR1 site on 5`-end	Construction of 122S1
T1 F-R	TTTGCAGACTACGGGCCTAAACAAAAGGCCGAACACATGT	Intergenetic region (2015413-2016412) with EcoR1 site on 5`-end	Construction of 122S1
T1sacB-F	ACATGTGTTCGGCCTTTTGTTTAGGCCCGTAGTCTGCAAA	*sacB* with 40 nucleotides	122S1 construction/*sacB* PCR
T1T3 sacB-R	CCGAACAGGCTTATGTCCACACCCATCGGCATTTTCTTTTG	*sacB* with 40 nucleotides	122S1 construction/*sacB* PCR
T1T3 aadA-F	CAAAAGAAAATGCCGATGGGTGTGGACATAAGCCTGTTCGG	*aadA* with 41 nucleotides	Construction of 122S1
T1aadA-R	GAAGTTGAATTGGACCCGTTAGTGCATCTAACGCTTGAGT	*aadA* with 41 nucleotides	Construction of 122S1
T1 R-F	ACTCAAGCGTTAGATGCACTAACGGGTCCAATTCAACTTC	Intergenetic region (2016454-20175697) with Sal1 site	Construction of 122S1
T1 R-R	TCAGTCGACCTCACGAAAGAACCCGACTT	Intergenetic region (2016454-20175697) with Sal1 site	Constructing 122S1
ISRj2_F	GCTCCGGGTCACAAGATCTA	ISRj2, internal region	IS-mediated deletion
ISRj2_R	GCCCTGATCCGTGTTGAAAA	ISRj2, internal region	IS-mediated deletion
ISRj1_F	TGTCCAATATGTCTGGCGGT	ISRj1, internal region	IS-mediated deletion
ISRj1_R	CCGCTTGTAATCGTGGCTTT	ISRj1, internal region	IS-mediated deletion
ISBj2_F	GACGATGGTGCGCAATCTTC	ISBj2, internal region	IS-mediated deletion
ISBj2_R	ATCCTGGCCTGGAGTCAGAT	ISBj2, internal region	IS-mediated deletion
ISBj12-F	GTGCTCACCGACATTGCTAC	ISBj12, internal region	IS-mediated deletion
ISBj12-R	GAACCTACGATAGCCGACCA	ISBj12, internal region	IS-mediated deletion
ISFK1 1081-F	ATCGCCACGAGAATTTGCT	ISFK1, internal region	IS-mediated deletion
ISFK1 1081-R	CCCCAATCATCCAAAATCAG	ISFK1, internal region	IS-mediated deletion
Fix-del_F1	CAGGCGGCATTCTCTTCATC	β2 in USDA122, upstream region	IS-mediated deletion
Fix-del_F2	CAGGCGGCATTCTCTTCATC	α2 in USDA122, upstream region	IS-mediated deletion
Fix-del_F3	ACGAGCTTGCAATGCGTTAT	β3 in USDA122, upstream region	IS-mediated deletion
Fix-del_F4	AACTTGTTCGCCCATTTCGG	α5 in USDA122, upstream region	IS-mediated deletion
Fix-del_F5	AATAGAGGTCGAGGAAGGCG	α7 in USDA122, upstream region	IS-mediated deletion
Fix-del_F6	TCCCATTGAGTGAAGCCGAC	α3 in USDA122, upstream region	IS-mediated deletion

Fix-del_F7	TGACCCTTGACGAGTTGACG	α4 in USDA122, upstream region	IS-mediated deletion
Fix-del_F8	GACTTGAGCGACGAAATCGC	α6 in USDA122, upstream region	IS-mediated deletion
Fix-del_F11	CGTACAAAGTTTTGGCGCCA	β3 in USDA122, upstream region	IS-mediated deletion
Fix-del_F15	ATGGTATCACGACCGATGGC	β4 in USDA122, upstream region	IS-mediated deletion
Fix-del_R1	GCAATAAGGTGGGAGCGATG	β3 in USDA122, downstream region	IS-mediated deletion
Fix-del_R2	CCTTGCGTCTCGAGTTTAGC	α5 in USDA122, downstream region	IS-mediated deletion
Fix-del_R3	GGTCCATGTGAATGACGACG	α7 in USDA122, downstream region	IS-mediated deletion
Fix-del_R4	CAAATCCCGCCAATCCGTAC	β2 in USDA122, downstream region	IS-mediated deletion
Fix-del_R5	CGACAAGTTCATGAGCGAGG	α2 in USDA122, downstream region	IS-mediated deletion
Fix-del_R6	TCGCCGCTTGTTGAACAAAG	α6 in USDA122, downstream region	IS-mediated deletion
Fix-del_R8	TTGTACATGCCGAGGGACAC	α3 in USDA122, downstream region	IS-mediated deletion
Fix-del_R9	CAGCCGCTAGACCGTCTTAG	α4 in USDA122, downstream region	IS-mediated deletion
Fix-del_R11	CGAGTGGATCTACGACAGCC	β3 in USDA122, downstream region	IS-mediated deletion
Fix-del_R15	TGGAAGGTGTGGACGCTTAC	β4 in USDA122, downstream region	IS-mediated deletion
Fix-del_F1D	TCCCATTGAGTGAAGCCGAC	α3D in USDA110, upstream region	IS-mediated deletion
Fix-del_F2D	AACACTTGGGTCCGCATGAT	α2D in USDA110, upstream region	IS-mediated deletion
Fix-del_F3D	ACGGAACAGTGCTCATTCGT	α4D in USDA110, upstream region	IS-mediated deletion
Fix-del_F4D	TCCACATTGAAGGCTCGTCC	α5D in USDA110, upstream region	IS-mediated deletion
Fix-del_F5D	GTAGAAGACGCAGAGCAGCT	α6D in USDA110, upstream region	IS-mediated deletion
Fix-del_R1D_F3J	CAGCCGCTAGACCGTCTTAG	α4D in USDA110, downstream region, α4J in J5, upstream region	IS-mediated deletion
Fix-del_R2D	ATACGACGAGCTAACTGCCG	α5D in USDA110, downstream region	IS-mediated deletion
Fix-del_R3D	CGGCCGAGGTCGTAAAACTA	α6D in USDA110, downstream region	IS-mediated deletion
Fix-del_R4D	TTCACCAGCCTCGAGTTCAC	α3D in USDA110, downstream region	IS-mediated deletion
Fix-del_R5D	GAAGATTGCGCACCATCGTC	α2D in USDA110, downstream region	IS-mediated deletion
Fix-del_F1J	CTTGAGCGCCGAAACTATGC	α3J in J5, upstream region	IS-mediated deletion
Fix-del_F2J	GATTCCATCGGCCAGAACGA	γ6J in J5, upstream region	IS-mediated deletion
Fix-del_F4J	GCTCGGTACTTCATCCTCCG	γ9J in J5, upstream region	IS-mediated deletion
Fix-del_R1J	TGACCCTTGACGAGTTGACG	α4J in J5, downstream region	IS-mediated deletion
Fix-del_R2J	ATGAAGCTGCCCATCAACGA	γ9J in J5, downstream region	IS-mediated deletion
Fix-del_R3J	CTCCTCGAAGATCGTGACCG	a3J in J5, downstream region	IS-mediated deletion
Fix-del_R4J	GGATCCTGATCGATGGGCAG	γ6J in J5, downstream region	IS-mediated deletion
ISBj2 F-1	TGGCTCAATCAGGTCGAAGT	γ3 in USDA122, upstream region	IS-mediated deletion
ISBj2 R-1	TGCCGCTTGATGTCAGAGTA	γ5 in USDA122, downstream region	IS-mediated deletion
ISBj12 F-1	GATCGATCGCCTTGTTCACC	δ1 in USDA122, upstream region	IS-mediated deletion
ISBj12 R-1	GGAAGCGCTTATCCCTACCA	δ2 in USDA122, downstream region	IS-mediated deletion

ISFK1 F-3	CACAACACCCCAATGACGTG	ε5 in USDA122, upstream region	IS-mediated deletion
ISFK1 R-2	GATCTTCGAACTATAGCGCGA	ISFK1, internal region	IS-mediated deletion
WA08 del_F	CCCTTGTCCTCAGCATCGAT	εX in USDA122, upstream region	IS-mediated deletion
ISFK1 R-1	ATCACGTCTTCGACCTCTGG	ε9 in USDA122, downstream region	IS-mediated deletion
a2M-F	AAAAGCTGCCCAATGACTCG	α2 in USDA122, upstream region	IS-mediated deletion
a5M-R	CCTTGCGTCTCGAGTTTAGC	α5 in USDA122, downstream region	IS-mediated deletion
a6M-R1	GACCTGTGGACCAAAAGCAG	α6 in USDA122, downstream region	IS-mediated deletion
a25 specific-F	AACTTCCGCGTTTTGTTCGA	Intergenic region (2083855-2084154) on USDA122 genome	Competition experiment
a25 specific-R	GCGTGACGACCTGATAAACC	Intergenic region (2083855-2084154) on USDA122 genome	Competition experiment
a25 a26 specific-F	TGGGTACTAGTCAGGCTCCT	Intergenic region (1907564-1908278) on USDA122 genome	Competition experiment
a25 a26 specific-R	TGCGATTGTAGATGGCGTTG	Intergenic region (1907564-1908278) on USDA122 genome	Competition experiment
BRsigA_F	CAACCTCCGTCTCGTGATCT	*sigA* in USDA122, internal region	Competition experiment
BRsigA_R	CGTAGGTCGAGAACTTGTAGCC	*sigA* in USDA122, internal region	Competition experiment
G4_F	TAGTCCGTCAGTCCCGTCAT	g4 in USDA110, upstream region	Genome duplication
G4_R	GCTCGGTACTTCATCCTCCG	g4 in USDA110, downstream region	Genome duplication
G8_F	CCGATACACATGCCGGATCA	g8 in USDA110, upstream region	Genome duplication
G8_R	GATGGAGATCCTCAAGCGGG	g8 in USDA110, downstream region	Genome duplication
G2_F	TTGGCTAACACGCTCGAACT	g2 in USDA110, upstream region	Genome duplication
G2_R	TATCTCGCCAATCTTCCGGC	g2 in USDA110, downstream region	Genome duplication
G7_F	CTTTGTTCAACAAGCGGCGA	g7 in USDA110, upstream region	Genome duplication
G7_R	GCATCGCACCTAGCACTACT	g7 in USDA110, downstream region	Genome duplication

References

1. M. A. Cole, G. H. Elkan, Transmissible resistance to penicillin G, neomycin, and chloramphenicol in *Rhizobium japonicum*. Antimicrob. Agents Chemother. **4**, 248-253 (1973).
2. J. H. Miller, A short course in bacterial genetics: a laboratory manual and handbook for *Escherichia coli* and related bacteria (CSHL Press, Cold Spring Harbor, NY., 1992).
3. S. Akao, H. Kouchi, Light microscopic observation of root hair curling of soybean induced by *Rhizobium* infection. *Jpn. J. Soil Sci. Plant Nut*r. **60**, 53–55 (1989).
4. Y. Ohtsubo, W. Ikeda-Ohtsubo, Y. Nagata, M. Tsuda, GenomeMatcher: a graphical user interface for DNA sequence comparison. BMC Bioinformatics **9**, 376 (2008).
5. T. Kaneko *et al.*, Complete genomic sequence of nitrogen-fixing symbiotic bacterium *Bradyrhizobium japonicum* USDA110. *DNA Research.* **9**, 189-197 (2002).
6. M. Sugawara *et al.,* Variation in bradyrhizobial NopP effector determines symbiotic incompatibility with *Rj2*-soybeans via effector-triggered immunity. *Nat. Commun.* **9**, 3139 (2018).
7. K. Kanehara, K. Minamisawa, Complete genome sequence of *Bradyrhizobium japonicum* J5, isolated from a soybean nodule in Hokkaido, Japan. *Genome Announc.* **5**, e01619-16 (2017).
8. R. R. Wick, L. M. Judd, C. L. Gorrie, K. E. Holt, Unicycler: Resolving bacterial genome assemblies from short and long sequencing reads. *PLoS Comput. Biol.* **13**, e1005595 (2017).
9. Y. Tanizawa, T. Fujisawa, E. Kaminuma, Y. Nakamura, M. Arita, DFAST and DAGA: Web-based integrated genome annotation tools and resources. *Biosci. Microbiota Food Health* **35**, 173–184 (2016).
10. T. Kaneko *et al.*, Complete genome sequence of the soybean symbiont *Bradyrhizobium japonicum* strain USDA6^{T}. *Genes (Basel).* **2**, 763–787 (2011).
11. T. Iida *et al.,* Symbiosis island shuffling with abundant insertion sequences in the genomes of extra-slow-growing strains of soybean bradyrhizobia. *Appl. Environ. Microbiol.* **81**, 4143-4154 (2015).
12. R. Ledermann *et al.* Stable fluorescent and enzymatic tagging of *Bradyrhizobium diazoefficiens* to analyze host-plant infection and colonization. *Mol. Plant Microbe*

Interact. **28**, 959-967 (2015).

13. M. Sugawara *et al.,* Complete genome sequence of *Bradyrhizobium diazoefficiens* USDA 122, a nitrogen-fixing soybean symbiont. *Genome Announc.* **5**, e01743-16 (2017).
14. A. Schäfer, A.Tauch, W. Jäger, J. Kalinowski, G. Thierbach, Small mobilizable multi-purpose cloning vectors derived from the *Escherichia coli* plasmids pK18 and pK19: selection of defined deletions in the chromosome of *Corynebacterium glutamicum*. *Gene* **145**, 69-73 (1994).
15. J. Frey, H. M. Krisch, Omega mutagenesis in gram-negative bacteria: a selectable interposon which is strongly polar in a wide range of bacterial species. *Gene* **36**, 143–150 (1985).
16. D. H. Figurski, D. R.Helinski, Replication of an origin-containing derivative of plasmid RK2 dependent on a plasmid function provided in *trans*. *Proc. Natl. Acad. Sci. U.S.A.* **76**, 1648-1652 (1979).
